# Mechanism Insight of Cell Death Signaling by Thymol Derivatives on Trypanosomatidae Protozoan Parasites

**DOI:** 10.3390/antibiotics14040383

**Published:** 2025-04-05

**Authors:** Amani Omrani, Meriam Ben Youssef, Ines Sifaoui, Eduardo Hernández-Álvarez, Carlos J. Bethencourt-Estrella, Isabel L. Bazzocchi, Hichem Sebai, Jacob Lorenzo-Morales, Ignacio A. Jiménez, José E. Piñero

**Affiliations:** 1 Instituto Universitario de Enfermedades Tropicales y Salud Pública de Canarias, Universidad de La Laguna, 38296 La Laguna, Tenerife, Spain; alu0101534855@ull.edu.es (A.O.); alu0101534848@ull.edu.es (M.B.Y.); isifaoui@ull.edu.es (I.S.); cbethene@ull.edu.es (C.J.B.-E.); jmlorenz@ull.edu.es (J.L.-M.); jpinero@ull.edu.es (J.E.P.); 2 Laboratory of Functional Physiology and Valorization of Bio-Ressources, Higher Institute of Biotechnology of Beja, University of Jendouba, Beja 382-9000, Tunisia; sebaihichem@yahoo.fr; 3 Instituto Universitario de Bio-Orgánica Antonio González, Departamento de Química Orgánica, Universidad de La Laguna, Avenida Astrofísico Francisco Sánchez 2, 38206 La Laguna, Tenerife, Spain; alu0100947311@ull.edu.es (E.H.-Á.); ilopez@ull.edu.es (I.L.B.); 4 Departamento de Obstetricia y Ginecología, Pediatría, Medicina Preventiva y Salud Pública, Toxicología, Medicina Legal y Forense y Parasitología, Universidad de La Laguna, C/Sta. María Soledad s/n, 38200 La Laguna, Tenerife, Spain; 5 Consorcio Centro de Investigación Biomédica en Red, Área de Enfermedades Infecciosas, Instituto de Salud (CIBERINFEC) Carlos III, Av. Monforte de Lemos 3-5, Pabellón 11, 28029 Madrid, Spain

**Keywords:** thymol derivatives, *Leishmania amazonensis*, *Trypanosoma cruzi*, structure–activity relationship, mechanism of action, drug-likeness

## Abstract

Leishmaniasis and Chagas disease are parasitic diseases considered to be among the most important neglected diseases, with implications for both developed and developing countries. Currently, there are no effective therapeutic treatments for these diseases due to challenges in drug administration, high toxicity, high costs, and drug resistance. In this study, a series of eleven thymol derivatives were designed, synthesized, and evaluated for their in vitro kinetoplastid activity against *Leishmania amazonensis* and *Trypanosoma cruzi*, as well as their cytotoxicity against a murine macrophage cell line. The most active compounds, thymol anysoate (**9**) and thymol picolinate (**10**), displayed the highest kinetoplastid activity with IC_50_ values of 22.87 and 25.16 µM against *L. amazonensis* and *T. cruzi*, respectively. Notably, both compounds demonstrated an excellent selectivity index against the mammal cell line. Structure–activity relationship studies revealed that the ester group plays a crucial role in activity. The most promising derivatives, **9** and **10**, activate autophagy and apoptosis-like processes in the treated cells. Atomic force microscopy observations showed that derivative **9** induces the formation of cytoplasmic vacuoles, indicating an autophagic process, and drug-likeness analysis revealed that it meets all the pharmacokinetic criteria. Overall, these results highlight derivative **9** as a potential lead compound for the development of new drugs for the treatment of Trypanosomatidae infections and warrants further studies to elucidate the cell death cascade involved.

## 1. Introduction

Neglected tropical diseases caused by bacteria, virus, helminth parasites, and protozoa [1] represent a serious global health problem, particularly in developing countries. Kinetoplastid protozoan parasites are a group of flagellated protozoa [2] characterized by a unique mitochondrial DNA structure located at the base of the flagellum, known as ‘kDNA’ [3]. These infections have a high prevalence but remain a low priority for investment [2]. Among unicellular protozoa, *Trypanosoma brucei*, *Trypanosoma cruzi*, and *Leishmania* parasites are responsible for African sleeping sickness, Chagas disease, and leishmaniasis, respectively [4].

Chagas disease, also known as American trypanosomiasis, is endemic in Latin America and causes approximately 12,000 deaths annually. Moreover, due to globalization, it has spread to other countries and continents [5]. The causative agent, *T. cruzi*, is a heteroxenous protozoan parasite that requires both mammalian hosts and triatomines to complete its life cycle [6]. This infection manifests itself in two phases: an acute phase, which is generally asymptomatic or may cause a self-limiting febrile illness. During this phase, therapy with antiprotozoal drugs such as benznidazole is effective [7]. However, 10 to 30 years after infection, 30–40% of patients develop chronic disease with cardiac, digestive, or cardiodigestive symptoms [7]. Moreover, the efficacy of therapy decreases significantly as the infection progresses and the patient ages [8]. Leishmaniasis is considered one of the most challenging health problems worldwide. According to the World Health Organization (WHO), this disease is endemic in more than 88 countries. Leishmaniasis is transmitted to humans through the bite of phlebotomine sandflies, and presents itself in various clinical forms, including cutaneous, diffuse cutaneous, mucocutaneous (espundia), visceral (kala-azar), post-kala-azar dermal leishmaniasis, and recidivans [9]. The prevalence of leishmaniasis has been documented as being approximately 12 million cases, with an annual incidence of 1.5–2 million cases of cutaneous leishmaniasis and 500,000 cases of visceral leishmaniasis, resulting in approximately 50,000 deaths each year [10]. The current treatment for leishmaniasis primarily relies on sodium stibogluconate, meglumine antimoniate, and pentamidine as first-line drugs. Second-line treatments such as miltefosine, paromomycin, or liposomal amphotericin B used alone or in combination have become the preferred options to prevent the emergence of resistance [11]. Current treatments for both Chagas disease and leishmaniasis are highly toxic to host cells and prone to resistance. Consequently, there is an urgent need to develop new agents that are both safe and effective.

In the field of drug discovery, particularly for anti-infective agents, natural products and medicinal plants have served as essential sources for developing new bioactive compounds since ancient times [12,13,14], including those with antiparasitic properties [15]. In particular, several studies have confirmed the pharmacological properties of thymol, a phenolic monoterpenoid [16], which exhibits analgesic, antispasmodic, antibacterial, antifungal, antioxidant, anti-inflammatory, and antitumoral activities [17]. Moreover, thymol has demonstrated significant efficacy against *Trypanosoma cruzi* and various *Leishmania* species. Specifically, thymol exhibits inhibitory activity against intracellular amastigotes of *T. cruzi*, comparable to standard treatments such as benznidazole. Likewise, thymol has shown potent leishmanicidal effects against both promastigote and amastigote forms of *Leishmania amazonensis* and *L. chagasi*. Furthermore, its high selectivity index and low cytotoxicity enhance thymol’s therapeutic potential, making its derivatives a promising focus for further research in the fight against parasitic diseases [18,19]. Despite its widespread use in the pharmaceutical sector, thymol remains underutilized due to its low aqueous solubility, strong odor, and intense taste [20]. To overcome these limitations and improve thymol’s pharmacological profile, a series of eleven ester derivatives were synthesized and evaluated against two kinetoplastid models: *Trypanosoma cruzi* and *Leishmania amazonensis*. The cell death mechanism of the most selective derivatives on clinically relevant cell types was also analyzed. Additionally, a preliminary structure–activity relationship (SAR) study and predicted ADME properties are discussed.

## 2. Results and Discussion

### 2.1. Chemistry

**Figure 1:**
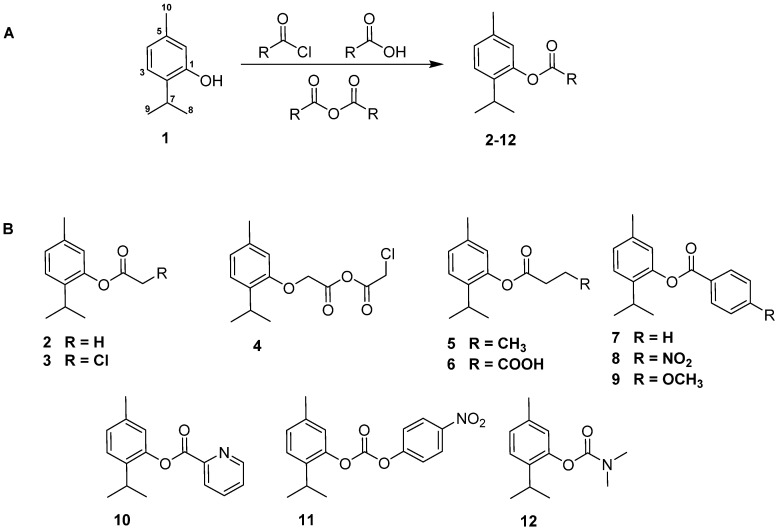
(**A**) Synthesis pathway of thymol derivatives. (**B**) Chemical structures of thymol derivatives **2**–**12**.

Natural products and their semisynthetic derivatives continue to serve as crucial sources of potential drug leads in medicinal chemistry. Indeed, there is increasing interest among organic chemists and biologists to investigate these compounds due to their use as excellent starting materials for derivative design and synthesis, often exhibiting enhanced biological activities compared to their natural counterparts [12,13]. In this context, the activity of thymol (**1**) prompted the initiation of a hit-to-lead program aimed at improving its kinetoplastid activity. Consequently, eleven thymol derivatives (**2**–**12**) were synthesized and evaluated against *Leishmania* and *Trypanosoma* parasites. To obtain the corresponding ester derivatives, thymol was esterified with different carboxylic acid derivatives (Figure 1).

The structures of the derivatives were elucidated based on their spectroscopic analysis, including ^1^H and ^13^C NMR spectra and high-resolution mass spectroscopy (see Supplementary Materials, Figures S1–S35). Their spectral data were compared with those reported in the literature for confirmation. Thus, the derivatives were identified as: thymyl acetate (**2**) [21], thymyl chloroacetate (**3**) [22], 2-thymyl-2′-(chloro)-acetic anhydride (**4**) [23], thymyl butyrate (**5**) [22], thymyl hemisuccinate (**6**) [23], thymyl benzoate (**7**) [21], thymyl-4-nitrobenzoate (**8**) [24], thymyl anysoate (**9**) [24], thymyl picolinate (**10**) [25] thymyl-4-nitrophenylcarbonate (**11**) [23], and thymyl *N*,*N*-dimethyl carbamate (**12**) [23].

### 2.2. In Vitro Kinetoplastid Activity on L. amazonensis and T. cruzi

Thymol (**1**) and its derivatives (**2**–**12**) were evaluated for their in vitro kinetoplastid activity against the promastigote stage of *L. amazonensis* and the epimastigote stage of *T. cruzi*. In addition, their safety profiles were assessed on a murine macrophage cell line J774.A1. The results of the antikinetoplastid assays (Table 1, see Supplementary Materials, Figures S48–S50) revealed that derivatives **9** and **10** were the most potent inhibitors of both parasites, with IC_50_ values of 29.1 and 34.4 µM, respectively, against *L. amazonensis*, and 26.8 and 35.5 µM against *T. cruzi*. Derivatives **9** and **10** also demonstrated significantly enhanced potency compared to the parent compound (**1**), exhibiting an 11- and 9-fold increase in potency against *L. amazonensis*, respectively. Similarly, both derivatives showed a 7- and 5-fold increase in potency against *T. cruzi*, respectively. Additionally, derivatives **3**, **4**, **7**, **8**, and **11** displayed moderate activity, with IC_50_ values ranging from 50.0 to 150.0 µM, slightly higher than the parent compound. By contrast, derivatives **5** and **12** exhibited weak activity, with IC_50_ values between 150.0 and 250.0 µM. The remaining derivatives **2** and **6** were inactive (IC_50_ > 350 µM).

Based on the IC_50_ values of thymol (**1**) and its derivatives (**2**–**12**) against the promastigote and epimastigote stages of *L. amazonensis* and *T. cruzi*, respectively. Derivatives **9** and **10** were selected for further evaluation against the intracellular stage of both protozoan models (Table 2).

Derivative **9** showed higher activity than derivative **10**, with IC_50_ values of 15.2 and 9.1 µM against the intramacrophage forms of *L. amazonensis* and *T. cruzi*, respectively. Furthermore, the selectivity index of derivative **9** against *L. amazonensis* was higher than that of miltefosine, the only orally approved drug currently available for the treatment of leishmaniasis [26].

These results are consistent with previous studies demonstrating that oregano essential oil exhibits significant in vitro and in vivo effects against *L. amazonensis* [27]. Additionally, the dichloromethane fraction from *Oliveira decumbens* has shown activity against *L. major* and *L. tropica* [28], both of which contain thymol as one of their main components. Notably, thymol has been reported as inactive against the promastigote stage of *L. major* and *L. tropica* [28], yet it has demonstrated activity against *L. infantum* (IC_50_ = 7.2 μg/mL) [29], *L. chagasi* (IC_50_ = 28.0 μg/mL) [30], and *L. amazonensis* (IC_50_ = 26.8 μg/mL) [31]. Moreover, thymol derivatives such as acetyl and benzoyl derivatives have shown promising activity against the promastigote (EC_50_ = 9.07 and 8.67 μg/mL, respectively) and amastigote (EC_50_ = 10.95 and 15.09 μg/mL, respectively) stages of *L. infantum* and *L. chagasi* [21]. Regarding trypanocidal properties, *Limonium oleofolium* essential oil, which contains thymol, has demonstrated its effectiveness [32]. Additionally, thymol carbonate derivatives have been reported as promising trypanocidal agents [33].

### 2.3. Preliminary Structure–Activity Relationship

**Figure 2:**
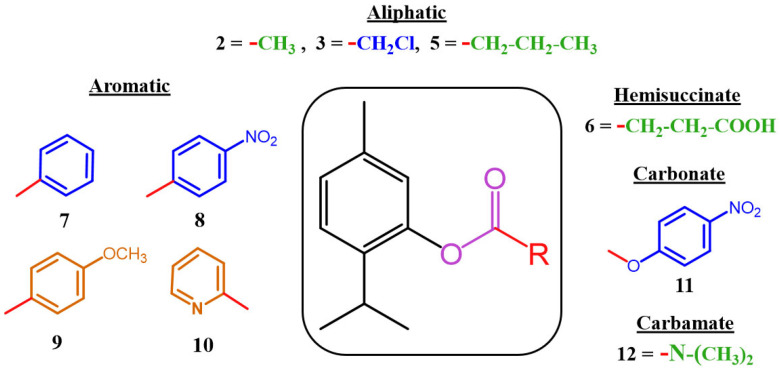
Preliminary structure–kinetoplastid activity relationship analysis of thymol derivatives. In orange (potent), blue (moderate), and green (inactive).

The influence of the substitution pattern of the tested ester derivatives on their antiparasitic activity was analyzed, revealing the following features (Figure 2): (a) The presence of an *O*-saturated aliphatic ester (**2**–**5**) enhanced activity against *L. amazonensis* and *T. cruzi* compared to the parent compound, thymol (**1**). A comparative analysis between derivatives **2** and **3** showed that while the acetate ester (**2**) was inactive against both parasites, derivative **3** was the most potent among the aliphatic derivatives. This suggests that the incorporation of a chlorine atom into the structure enhances the antiparasitic activity, contributing to the moderate potency of derivative **3**. (b) The presence of an *O*-aromatic ester (**7**–**10**) also improved antiparasitic activity compared to the parent compound, with derivatives **9** and **10** standing out as the most active against both parasites. This finding suggests that the electron-donating group in derivative **9** and the pyridine moiety in derivative **10** contribute to increased potency. (c) A comparative analysis between derivatives **5** and **6** showed that replacing a methyl group with a carboxylic acid one reduced activity, from moderate (**5**) to inactive in the hemisuccinate derivative (**6**). (d) The introduction of a carbonate group in derivative **11** or a carbamate group in derivative **12** increased activity compared to thymol (**1**), particularly in the case of derivative **11**.

In addition, the results of an SAR analysis, in accordance with previous works [21,33], clearly indicate that acylation enhances antiparasitic activity against both parasites. This suggests that the type of ester group plays a crucial role in determining the activity of this series of thymol derivatives.

Our results, along with previous studies, motivated us to further investigate the potential and mechanism of antikinetoplastid action of a series of thymol derivatives. These were synthesized to enhance their potency and the selectivity index of thymol against *L. amazonensis* and *T. cruzi*. Furthermore, an attempt to elucidate the mechanism of action of thymol derivatives **9** and **10** in both protozoan models. To do so, various image base fluorescence assays have been used to seek the effect of both molecules and cells feature including chromatin condensation, the cell’s permeability, and mitochondria function.

### 2.4. Mechanism of Action of Derivatives ***9*** and ***10***

**Figure 3:**
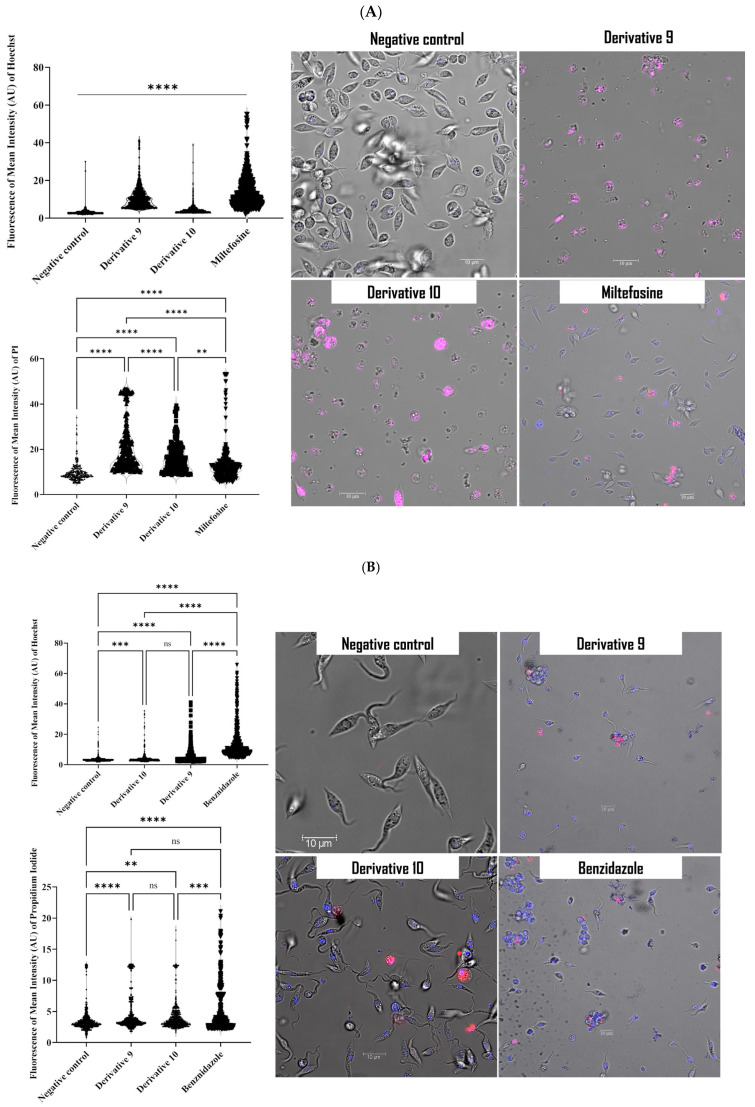
Evaluation of chromatin condensation using Hoechst/PI Staining after 24 h incubation with IC_90_ against *L. amazonensis* promastigotes (**A**) and *T. cruzi* epimastigotes (**B**). Images (63×) are representative of the observed cell population, obtained using a Leica SPE confocal microscopy. The violin plot depicts distributions of fluorescence intensities for each sample obtained by EVOS M5000 software. Differences between the values were assessed using one-way analysis of variance (ANOVA). ** *p* < 0.01; *** *p* < 0.001; **** *p* < 0.0001 and ns: non-significance.

In this study, a double staining assay was performed using Hoechst and PI dyes to determine the type of cell death induced by both derivatives, **9** and **10**, in *L. amazonensis* and *T. cruzi*. After 24 h of incubation with the corresponding IC_90_ of each derivative, the fluorescence emitted by treated and untreated cells was measured using an image-based cytometer, EVOS M5000. The results are presented as a violin plot to depict the differences in fluorescence distribution between treated and untreated cells (Figure 3).

For both staining procedures, treated cells have a much more elongated distribution compared to the negative control. Among treated cells, one-way ANOVA analysis revealed that derivative **9** induced higher fluorescence intensities for both Hoechst and PI staining, reflecting a higher percentage of cells undergoing late stages of apoptosis. Consequently, derivatives **9** and **10** appear to trigger apoptosis by increasing the chromatin condensation.

**Figure 4:**
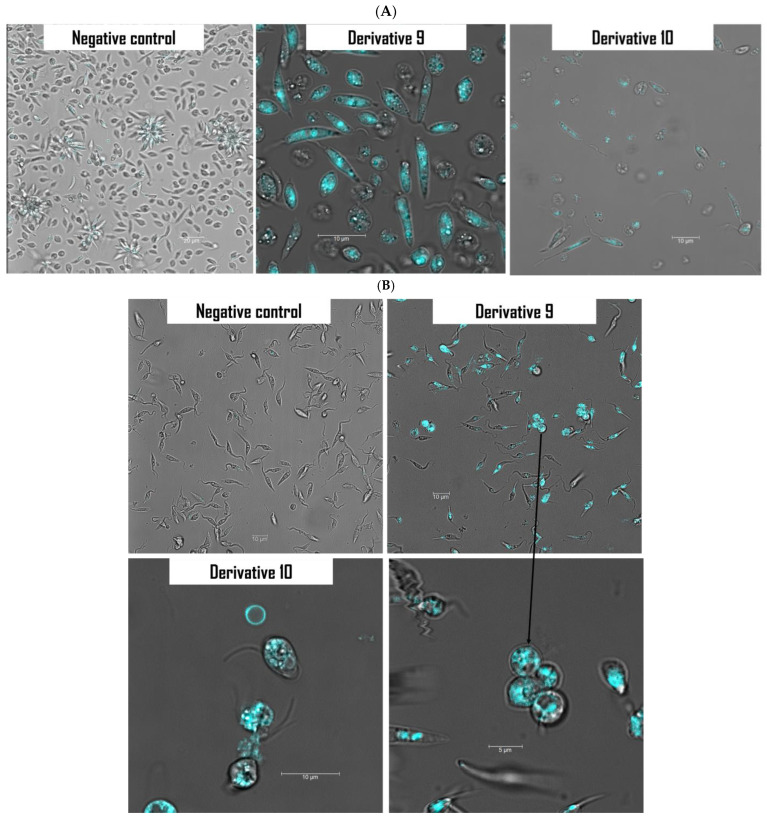
Induction of autophagy by derivatives **9** and **10** in *L. amazonensis* promastigotes (**A**) and *T. cruzi* epimastigotes (**B**) using monodansylcadaverine staining. Cells were observed under a Leica SPE confocal microscopy.

Monodansylcadaverine (MDC) staining was used to check on the presence of autophagic vacuoles. This dye has autofluorescence that stains the lipid component of the autophagy membrane. After 24 h of treatment with both derivatives (**9** and **10**), cells were viewed under a confocal microscope (Figure 4). The MDC-labeled autophagic vacuoles appeared as distinct punctuate structures in the cytoplasm.

**Figure 5:**
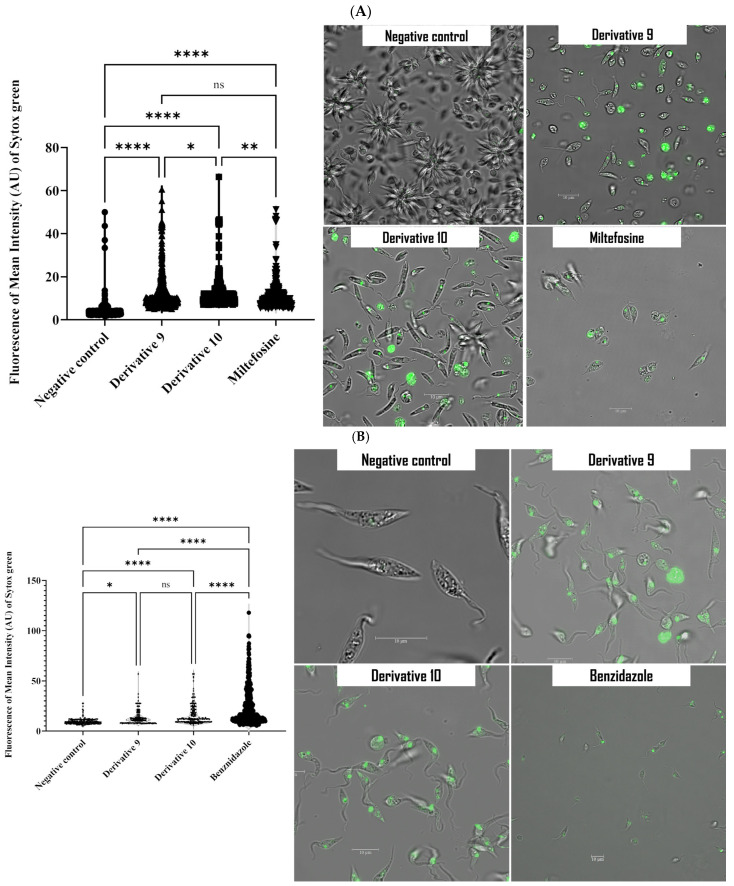
Detection of plasma membrane permeability in *L. amazonensis* (**A**) and *T. cruzi* (**B**) Using SYTOX^®^ Green staining after 24 h incubation with Derivatives **9** and **10**. Images (63×) are representative of the cell population observed in the performed experiments. Images were obtained using a Leica SPE confocal microscopy. The violin plot depicts distributions of fluorescence intensities for each sample obtained by EVOS M5000 software. Differences between the values were assessed using one-way analysis of variance (ANOVA). * *p* < 0.05; ** *p* < 0.01; **** *p* < 0.0001 and ns: non-significance.

The SYTOX^®^ Green staining, a DNA specific stain, was used to study the effect of derivatives **9** and **10** on membrane permeability in cells. The fluorochrome enters cells with compromised membrane permeability and emit green fluorescence upon binding to DNA [34]. After 24 h of treatment, the fluorescence intensity was measured using the EVOS M5000. The resulting fluorescence intensities are presented in violin plot. As shown in Figure 5, derivative **10** caused greater damage to membrane permeability in both parasites, as indicated by higher green fluorescence compared to derivative **9**.

**Figure 6:**
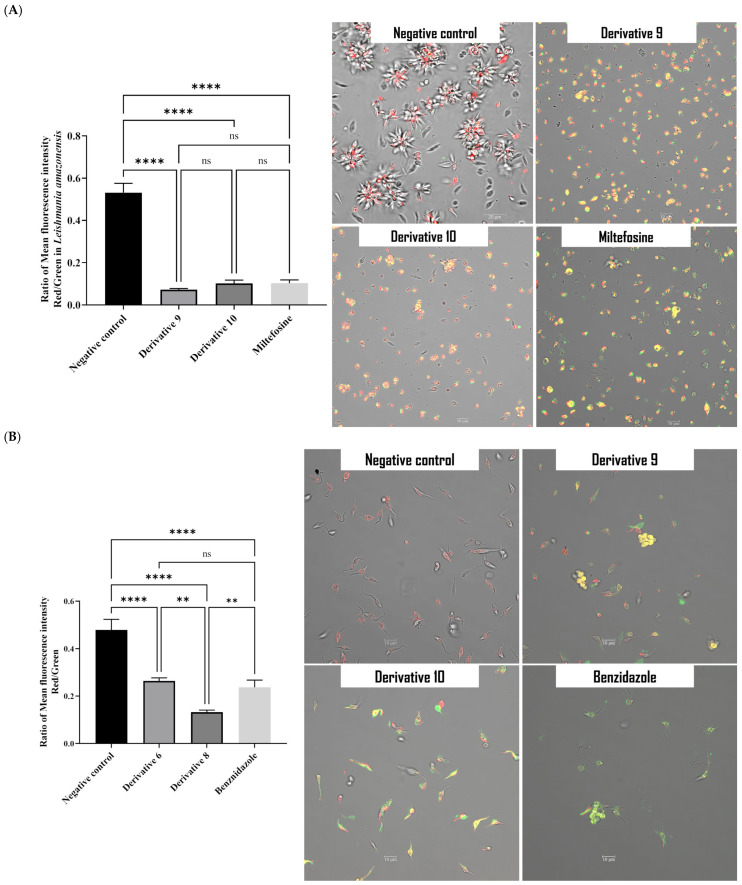
(**A**). Detection of mitochondrial membrane potential in *L. amazonensis* promastigotes using JC-1 staining after 24 h incubation with derivatives **9** and **10**. Images (63×) are representative of the cell population observed in the performed experiments. Images were obtained using a Leica SPE confocal microscopy. The histogram graphs depict the ratio of mean fluorescence obtained by an Enspire plate reader. A Tukey test was conducted to compare the mean between different samples. (**B**). Detection of mitochondrial membrane potential in *T. cruzi* epimastigotes using JC-1 staining after 24 h incubation with derivatives **9** and **10**. Images (63×) are representative of the cell population observed in the performed experiments. Images were obtained using a Leica SPE confocal microscopy. The histogram graphs depict the ratio of mean fluorescence obtained by an Enspire plate reader. A Tukey test was conducted to compare the mean between different samples. ** *p* < 0.01; **** *p* < 0.0001 and ns: non-significance.

To study the effect of both derivatives on the mitochondria, three assays were conducted: the mitochondrial membrane potential using the JC-1 dye, the ATP level, and the intracellular ROS level. The mitochondria constitute the principal organelle to produce intracellular energy and to define the type of cell death occurring in the cells. JC-1 was used to differentiate between healthy and damaged mitochondria. After 24 h incubation with derivative **9**, an increase in green fluorescence was observed, reflecting the inability of JC-1 to form aggregates due to the low ΔΨm. The mean fluorescence for both forms of JC-1 (aggregate and monomeric) was determined using the EVOS M5000 software and the ratio of red fluorescence to green fluorescence was calculated. As shown in the histogram, the potential of the mitochondrial membrane of *L. amazonensis* treated with derivative decreased by 80% compared to untreated cells. Moreover, in *T. cruzi*, derivative **9** was also able to decrease significantly the ΔΨm by 70%. Thus, derivative **9** led to higher depolarization of the mitochondrial membrane potential in *T. cruzi*, as confirmed by confocal microscopy (Figure 6A,B).

**Figure 7:**
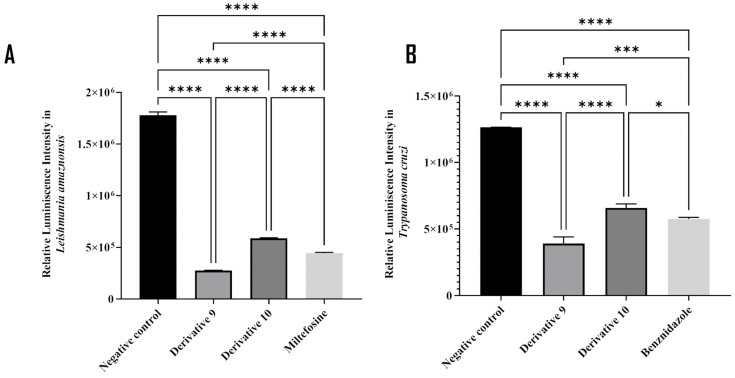
Results of the ATP levels of treated *L. amazonensis* (**A**) and *T. cruzi* (**B**) after 24 h of incubation with thymol derivatives **9** and **10**. A Tukey test was performed with the GraphPad.PRISM^®^ 9.0.0 software. * *p* < 0.05; *** *p* < 0.001; **** *p* < 0.0001.

The collapse of the mitochondrial membrane potential can lead to the inhibition of ATP production. In the assay, a luminescence-based method was used to measure the level of ATP produced by both treated and untreated kinetoplastids. As shown in Figure 7, derivatives **9** and **10** decreased ATP production by 84% and 67% in *L. amazonensis* (Figure 7A), and by 65% and 50% in *T. cruzi* (Figure 7B), compared to the negative controls.

**Figure 8:**
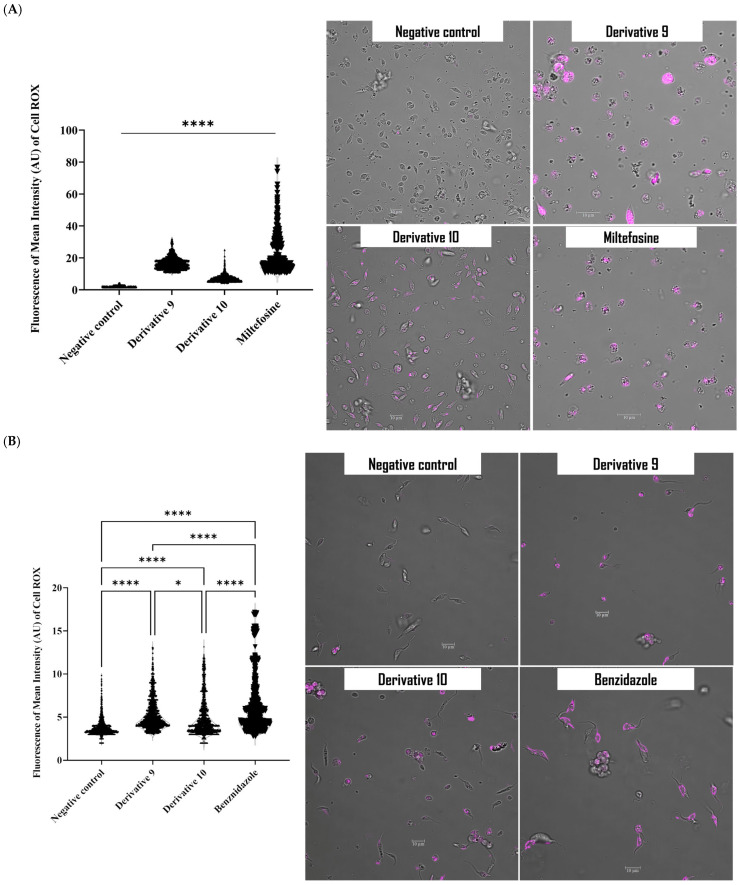
Oxidative stress assays of derivatives **9** and **10** in *L. amazonensis* (**A**) and *T. cruzi* (**B**), showing yellow fluorescence indicative of intracellular accumulation. Images (63×) are representative of the cell population observed in the performed experiments. The violin plots were obtained using an EVOS M5000 Cell Imaging System, Life Technologies, Spain. * *p* < 0.05; **** *p* < 0.0001.

Damaging mitochondrial function would affect the redox state of the cells, resulting in an imbalance between free radicals and antioxidant levels. The levels of intracellular reactive oxygen species (ROS) were measured after 24 h of incubation using CellROX Deep Red™. As shown in the histogram (Figure 8), treatment noticeably increased the emitted fluorescence, indicating that derivative **9** induced oxidative stress in *L. amazonensis* and *T. cruzi* by increasing ROS.

**Figure 9:**
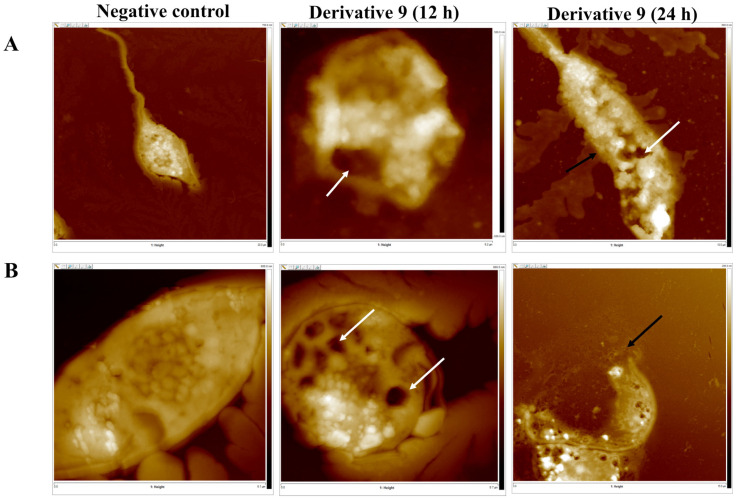
Morphological damage observed by atomic force microscopy on *L. amazonensis* (**A**) *and T. cruzi* (**B**) treated with compound **9** after 12 and 24 h. The cytoplasmic vacuoles are indicated with a white arrow while the damage induced in the cytoplasmic membrane is indicated with a black arrow.

The morphological effects of derivative 9 on *L. amazonensis* and *T. cruzi* were studied using atomic force microscopy (AFM). Both parasites were treated for 12 and 24 h with the IC_90_ concentration, then centrifuged and resuspended in a physiological serum. The results show representative AFM images of the treated cells, as well as those of the untreated control cells (Figure 9). Derivative **9** induced the formation of giant vacuoles in the cytoplasm of both kinetoplastids. Various cavities of different sizes were observed, with the largest measuring over 1 µm in diameter and up to 300 nm deep. Additionally, several smaller cavities of around 1 µm in diameter and 250–260 nm deep as well as others of approximately 300 nm in diameter and 240–250 nm deep were seen (white arrow). Notably, after 12 h of treatment, the edges of the membrane were clearly visible, whereas after 24 h, the damage progressed in depth, leading to membrane destruction (black arrow).

Overall, our findings suggest that both derivatives could induce programmed cell death via apoptosis and autophagic pathways. To date, the relationship between autophagy and apoptosis remains complex and not fully understood [35]. Depending on the type of cell and environmental conditions, autophagy may act as an agonist or antagonist to apoptosis [35]. In this study, derivatives **9** and **10** primarily affect the mitochondria, as well as other cellular compartments, notably the nucleus and plasma membrane. Based on confocal microscopy observations, thymol derivative **9** was selected for atomic force microscopy (AFM) analysis. The AFM images of treated epimastigotes and promastigotes revealed significant changes to the parasite’s cell surface and the presence of various cytoplasmic vacuoles. These microstructures, consistent with MDC staining, are likely to be autophagic vacuoles or autophagosomes. Previous studies have reported that the cytoplasm can become overwhelmed by autophagic and empty vacuoles, disturbing cellular membrane homeostasis and leading to membrane dysfunction [36,37]. Based on these observations, our findings suggest that derivative **9** inhibits both kinetoplastid models through a combination of autophagy and apoptosis. However, it remains unclear whether the programmed cell death in the parasites is induced by a combination of autophagy and apoptosis, or if autophagy-mediated cell death triggers apoptosis [38]. Further assays will be required to confirm the specific type of cell death involved in this study, including the assessment of protein expression such as ATG8.

### 2.5. Pharmacokinetic and Drug-Likeness Properties of Derivatives **9** and **10**

Furthermore, to effectively develop safe agents, it is crucial to assess their pharmacokinetic and bioavailability profiles while minimizing toxicity risks. In this regard, Swiss ADME software [39] was used to predict drug-likeness and pharmacokinetic properties, shedding light on essential characteristics such as oral bioavailability, permeability, solubility, oral absorption, and metabolism. The derivatives demonstrated a favorable drug-likeness profile and met the key pharmacokinetic criteria (see Supplementary Materials, Figures S36–S47 and Table 3). Analysis of important pharmacokinetic parameters, including hydrogen bond acceptors, hydrogen bond donors, topological polar surface area, lipophilicity, molecular weight, and rotatable bonds, revealed that the most active derivatives, thymyl anysoate (**9**) and thymyl picolinate (**10**), exhibited excellent phytochemical properties and lipophilicity [40]. Moreover, this study demonstrated that all derivatives exhibited an excellent ADME profile (see Supplementary Materials, Figures S25–S36 and Table 4).

In particular, the selected derivatives **9** and **10** displayed outstanding drug-likeness and medicinal chemistry properties. Their pharmacokinetic profiles indicated high gastrointestinal absorption and good blood-brain barrier permeability. Notably, none of the derivatives were identified as P-gp substrates. Regarding metabolism, both derivatives exhibited inhibitory activity against all tested cytochrome P450 isoenzymes (CYP1A2, CYP2C19, CYP2C9, CYP2D6, and CYP3A4), except for CYP3A4, where neither derivative showed inhibition, and CYP2C9, where derivative **10** was not an inhibitor.

**Figure 10:**
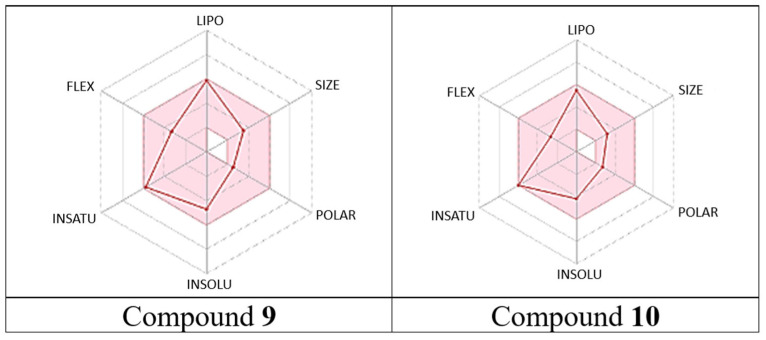
Bioavailability radar profile of derivatives **9** and **10**. LIPO: Lipophilicity. SIZE: Size. POLAR: Polarity. INSOLU: Insolubility. INSATU: insaturation. FLEX: flexibility. The pink area represented the optimal range of every descriptor.

The bioavailability radar also played a crucial role in evaluating the drug-likeness properties based on six descriptors: lipophilicity, size, polarity, solubility, unsaturation, and flexibility; each descriptor had an optimal range. In this regard, derivatives **9** and **10** demonstrated excellent bioavailability, as they met all the criteria and were positioned within the pink zone of the radar (Figure 10).

## 3. Material and Methods

### 3.1. General Procedures

^1^H NMR and ^13^C NMR were recorded at 300 K on Bruker Advance 500 (500 MHz for ^1^H-NMRand 125 ^13^C-NMR) and 600 (600 MHz for ^1^H-NMRand 150 ^13^C-NMR) NMR spectrometers. Chemical shifts were reported in δ (ppm), while coupling constants were expressed in Hz, with reference to the residual deuterated solvent (CDCl_3_: δ_H_ 7.26, δ_C_ 77.16), and TMS was utilized as an internal reference. For the ^1^H NMR experiments, the relaxation delay was 90° pulse, spectral width of 5500 Hz, and 32 k data points. The Gaussian function was applied to enhance the spectral resolution using −0.4 and 0.9 for Lorentzian broadening and Gaussian broadening, respectively. For the ^13^C NMR experiments, the corresponding parameters were 30° pulse, 21,000 Hz, and 62 k data points, and 3.0 s of relaxation delay. ESIMS and HRESIMS (positive mode) analyses were conducted using an LCT Premier XE Micromass Autospec spectrometer. Column chromatography was performed using silica gel 60 (particle sizes 15–40 and 63–200 µm), and for analytical and preparative TLC, we used Polygram Sil G/UV254 plates (Panreac, Barcelona, Spain). Reaction progress was monitored by TLC, and visualization of spots was achieved through UV light exposure and heating of silica gel plates sprayed with H_2_O-H_2_SO_4_-AcOH (1:4:20). Unless specified otherwise, solvents and reagents were procured from commercial suppliers and used without further purification. Analytical-grade solvents from Panreac and reagents from Sigma Aldrich (St Louis, MO, USA) were employed. Thymol (**1**), used as starting material, was sourced from Panreac (Barcelona, Spain).

### 3.2. General Procedure of Derivatives Synthesis ***2***–***12***

An excess of the corresponding carboxylic acid derivative was added to a mixture of compound **1**, dry triethylamine (Et_3_N), and a catalytic amount of 4-(dimethylamino)-pyridine (DMAP) in dry dichloromethane (CH_2_Cl_2_, 1 mL). The reaction progress was monitored by TLC using a hexane-CH_2_Cl_2_ (5:5) system. After concentration to dryness under reduced pressure, the residue was purified by column chromatography (CC) on silica gel, using hexane-CH_2_Cl_2_ mixtures of increasing polarity (7:3 to 5:5) as the eluent, affording the corresponding derivative. Their structures were confirmed by NMR spectroscopy (see Supplementary Materials, Figures S1–S24).

#### 3.2.1. Preparation of Derivatives **2**–**7** and **11**–**12**

These derivatives were synthesized following the present procedure [23].

#### 3.2.2. Preparation of Derivative 8

A mixture of **1** (30.2 mg, 0.2 mmol), Et_3_N (4 drops), DMAP (4.0 mg), and 4-nitrobenzoyl chloride (40.0 mg, 0.2 mmol) in CH_2_Cl_2_, was stirred at room temperature for 2 h. The residue was purified by CC, yielding derivative **8** (55.9 mg, 18.7%).

#### 3.2.3. Preparation of Derivative **9**

A mixture of **1** (30.5 mg, 0.2 mmol), Et_3_N (6 drops), DMAP (4.5 mg), and anisoyl chloride (0.1 mL, 0.7 mmol) in CH_2_Cl_2_, was stirred at room temperature for 2 h. The residue was purified by CC, yielding derivative **9** (18.3 mg, 6.4%).

#### 3.2.4. Preparation of Derivative **10**

A mixture of **1** (30.0 mg, 0.2 mmol), Et_3_N (4 drops), DMAP (4.5 mg), N,N-dicyclohexylcarbodiimide (5 mg), and picolinic acid (35.1 mg, 0.3 mmol) in CH_2_Cl_2_, was stirred at room temperature for 2 h. The residue was purified by CC, yielding derivative **10** (2.9 mg, 1.1%).

### 3.3. Biological Activity

#### 3.3.1. In Vitro Effect on Promastigote Stage of Leishmania amazonensis

The assay was conducted using the alamarBlue^®^ method as previously described [41]. Briefly, promastigotes of *L. amazonensis* were grown at 26 °C in Schneider’s medium (US Biological, Life sciences) and supplemented with 10% heat-inactivated fetal bovine serum and 0.04% of sodium bicarbonate. In a 96-well microtiter plates (Corning™, Corning, NY, USA) a serial dilutions of thymol derivatives were first prepared, and later, 10^6^ parasites/mL were added into each well. Subsequently, 10% of alamarBlue^®^ was added to the entire plate. The plates were incubated for 72 h at 26 °C.

#### 3.3.2. In Vitro Effect on Epimastigotes Stage of *Trypanosoma cruzi*

In a 96-well plate, a serial dilution of the tested derivatives was performed in liver infusion tryptose medium supplemented with 10% heat inactivated fetal bovine serum. Later, 100 µL of 5 × 10^5^ cells/mL of epimastigotes was added to each well. Afterwards, 20 µL of alamarBlue^®^ was added to each well, and the plate was maintained at 26 °C for 72 h.

#### 3.3.3. In Vitro Assay on the Intramacrophagic Stage

The most active molecules against promastigotes of *L. amazonensis* and epimastigotes of *T. cruzi* were evaluated against the intramacrophagic stage of both models as previously described [42,43]. Briefly, in a 96-well plate, macrophages from the J774.A1 cell line were seeded at a final concentration of 2 × 10^5^ cells/mL in Dulbecco’s Modified Eagle Medium (DMEM) and maintained at 37 °C in a 5% CO_2_ atmosphere until fully adhesion. Cells were infected either with L. amazonensis promastigotes (at a ratio of 1:10) or epimastigotes of T. cruzi (at a ratio of 1:7) (macrophage: parasite) and incubated at 37 °C in 5% CO_2_ for 24 h for Leishmania and 48 h for Trypanosoma. After infection, plates were washed using DMEM to eliminate external parasites. Subsequently, infected macrophages were treated with different concentrations of active compounds for 24 h. Later, macrophages were lysed using 30 μL of medium, Schneider for Leishmania, and in liver infusion tryptose for Trypanosoma, containing 0.05% of sodium dodecyl sulfate (SDS) for 30 s under agitation. Afterwards, 170 μL of the appropriated medium was derivatives added to each well to give a final volume of 200 μL. Subsequently, 10% of alamarBlue^®^ was added into each well and the plate was incubated at 26 °C for 72 h.

#### 3.3.4. Cytotoxicity Assay on Murine Macrophages

Cytotoxicity effect of the tested derivatives was conducted using macrophages of the murine cell line J774.A1 (American Type Culture Collection #TIB-67, city, country), as previously described [42]. The cells were maintained in DMEM at 37 °C in a 5% CO_2_ humidified incubator. All the plates containing alamarBlue^®^ were analyzed using a plate reader EnSpire Multimode Plate Reader^®^ at excitation and emission wavelengths of 570 and 585 nm, respectively (PerkinElmer, ThermoFisher Scientific, Madrid, Spain). The IC_50_ (concentrations able to inhibit 50% of parasites), IC_90_ (concentrations able to inhibit 90% of parasites), and CC_50_ (concentration able to inhibit 50% of murine macrophages) values were determined for each compound by nonlinear regression using GraphPad software.

### 3.4. Mechanism of Action Elucidation

The effect of derivatives **9** and **10** to modulate cell features was studied in the tested protozoa parasites, using various specific commercial kits. Parasite cells were incubated with the derivative at the previously calculated IC_90_ for 24 h. Subsequently, cells were washed and submitted to different treatments as mentioned by the manufacturer’s instructions. In all the assays, a plate reader EnSpire Multimode Plate Reader^®^ and/or an image base system fluorescence microscope EVOS M5000 were used for the fluorescence intensity quantification. For each protocol, images of representative population cells were obtained using an inverted confocal microscope Leica DMI 4000 B with LAS X software, and Leica HC PL APO 63×/1.40 OIL CS2 Objective (Barcelona, Spain).

#### 3.4.1. Double-Stain Assay for Programmed Cell Death (PCD) Determination

To identify the type of cell death occurring in both kinetoplastids after treatment, Hoechst 33342 and propidium iodide (PI) dyes were used. Even though both dyes are DNA affine, the Hoechst 33342 stain is cell permeable and known to distinguish apoptotic cells from healthy or necrotic cells [44], whereas the PI is cell impermeable and stain dead cells as late apoptotic or necrosis. After treatment, three types of staining could be observed: blue fluorescence for cells undergoing apoptosis, violet color as the merge of blue and red fluorescence for late apoptotic cells, and red color for dead cells.

#### 3.4.2. Labeling of Autophagic Vacuoles Using Monodansylcadaverine (MDC) Staining

The present method consists of staining the autophagic vacuoles with the treated cells by ion trapping and specific interactions with the vesicle membrane lipids. The experiment was carried out by following the manufacturer’s recommendations, and as described previously [45]. After 24 h of incubation, cells were stained with MDC reagent at 5 mM and incubated at 26 °C for 30 min in µ-Slide 8 (Ibidi, Gräfelfing, Germany). Later, the cells were washed with PBS and observed with Leica DMI 4000 B.

#### 3.4.3. Plasma Membrane Permeability

The SYTOX™ Green (Life Technologies, Madrid, Spain), a cell impermeable DNA binding stain, was used to evaluate the membrane permeability and integrity. The assay was conducted as detailed in the manufacture instructions, and as previously described [46].

#### 3.4.4. Analysis of the Mitochondrial Function

To evaluate the damage caused by the tested derivatives on the mitochondrial function, mitochondrial membrane potential, adenosine triphosphate (ATP) level, and reactive oxygen species (ROS) were measured as follows.

The measurement of mitochondrial membrane potential was carried out using the JC-1 assay. JC-1 is a mitochondria-specific stain whose fluorescence emission depends on the mitochondrial membrane potential. In healthy mitochondria, JC-1 aggregates and emits red fluorescence, whereas in unhealthy mitochondria, it remains in monomeric form and emits green fluorescence. Therefore, mitochondria with a collapsed membrane potential exhibit a low red-to-green fluorescence intensity ratio. The assay was performed according to the manufacturer’s instructions, as detailed in a previous study [46].

The adenosine triphosphate (ATP) level was measured using the CellTiter-Glo Luminescent Cell Viability Assay (Promega, Madrid, Spain) [46]. The mitochondria in which most cellular oxidations occur is considered the main source of cell’s ATP; any damage and/or malfunction of this organelle generate the depletion of ATP levels.

The measure of reactive oxygen species (ROS) was measured after treatment of 24 h using CellROX^®^ Deep Red fluorescent probe (Invitrogen, Madrid, Spain) [46]. The mitochondria constitute an essential organelle to neutralize the reactive oxygen species; the malfunction of this organelle would generate an imbalance between the antioxidants and the reactive oxygen.

#### 3.4.5. Atomic Force Microscopy (AFM) Analysis

Cultures of L. amazonensis and T. cruzi were incubated with derivative 9 at a final concentration equivalent to IC_90_ for 24 h. After incubation, cells were washed twice with PBS, and 10 µL of a 10^5^ cells/mL suspension was smeared onto glass slides. Prior to AFM analysis, samples were dried for 10 min. AFM topographic images were acquired in Peak Force mode using a multimode microscope equipped with a Nanoscope V control unit (Bruker). Scans were performed at rates of 0.5–1.2 Hz, employing FESP tips (50–100 kHz, 1–5 N/m) from Bruker. The damage caused by derivative **9** in both parasites was assessed by recording images at scales ranging from 100 µm × 100 µm to 0.6 µm × 0.6 µm, with a resolution of 512 points per line [47].

### 3.5. Statistical Analysis

All antiprotozoal tests were performed in triplicate, and results are presented as mean values ± standard deviation. Differences between values were assessed using one-way analysis of variance (ANOVA). Statistical significance was indicated as follows: *** *p* < 0.001; **** *p* < 0.0001; ns: not significant.

### 3.6. Swiss ADME Analysis

The pharmacokinetic and drug-like properties of thymol (**1**) and its derivatives (**2–12**) were estimated using Swiss ADME tools [39].

## 4. Conclusions

In summary, the initial hit, thymol, which exhibits biological activity in the micromolar range against *T. cruzi* and *L. amazonensis*, was investigated. The effect of esterification on its bioactivity was explored. In this study, we synthesized eleven derivatives using a structure-based design strategy to gain insights into this modification. Their antikinetoplastid structure–activity relationship analysis revealed that the acylation of thymol enhances its activity, with thymol anisate (**9**) and thymol picolinate (**10**) displaying the highest activity against both parasites. Furthermore, an ADME analysis was conducted to complement the hit-to-lead optimization process, showing that both derivatives possess favorable physicochemical and drug-likeness properties. The mechanism of action of compounds **9** and **10** in parasite inhibition was further investigated through fluorescence analysis, which indicated a positive regulation of the apoptosis and autophagy pathways. Through the esterification of thymol, we have successfully addressed several of its inherent disadvantages: volatility, odor, solubility, and lipophilicity. Moreover, compounds **9** and **10** offer notable advantages over the current treatment strategy: (a) Accessibility and cost-effectiveness: as derivatives from thymol, which is widely available and cheap, they surpass many synthetic treatments that require costly, complex precursors; (b) Synthetic simplicity: their synthesis via esterification is straightforward, scalable, and efficient, yielding high product amounts. This reduces production costs, simplifies quality control, and enables rapid scale-up for larger demands. The present research supports the potential of thymol as a source of lead compounds, enhancing our understanding of the antikinetoplastid therapeutic potential of naturally occurring phenolic monoterpenes targeting leishmaniasis and Chagas disease, and highlighting the need for further research in this field.

## Figures and Tables

**Table 1:** Kinetoplastid activity of thymol (**1**) and derivatives (**2**–**12**) against *L. amazonensis* promastigotes, *T. cruzi* epimastigotes, and cytotoxicity against murine macrophage cells.

Cmpds	*L. amazonensis* Promastigote	*T. cruzi* Epimastigote	Cytotoxicity	Selectivity *Leishmania*	Selectivity *Trypanosoma*
	IC_50_^a^	IC_50_^a^	CC_50_^b^	SI ^c^	SI ^c^
**1**	317.6 ± 21.4	206.0 ± 2.8	>300	>0.9	>1.5
**3**	78.8 ± 15.9	49.5 ± 3.2	>300	>3.8	>6.1
**4**	147.6 ± 10.0	123.7 ± 6.0	120.9 ± 0.4	0.8	0.9
**5**	216.5 ± 7.5	211.2 ± 7.3	>300	>1.4	>1.4
**7**	56.0 ± 5.6	66.0 ± 1.7	>300	>5.4	>4.5
**8**	76.4 ± 6.2	44.4 ± 2.5	>300	>3.9	>6.8
**9**	29.1 ± 3.8	26.8 ± 2.2	>300	>10.3	>11.2
**10**	34.4 ± 2.3	35.5 ± 2.5	254.5 ± 1.9	7.4	7.2
**11**	56.9 ± 7.2	66.9 ± 3.8	>300	>5.2	>4.5
**12**	228.6 ± 21.4	157.8 ± 2.9	>300	>1.3	>1.9
M ^d^	6.5 ± 0.3		72.2 ± 8.9	11.1	
B ^e^		6.9 ± 0.8	399.9 ± 1.4		58.0

^a^ IC_50_: Concentrations able to inhibit 50% of parasites expressed as µM ± standard deviation (SD). ^b^ CC_50_: Concentration able to inhibit 50% of murine macrophages expressed as µM ± standard deviation (SD). ^c^ SI: Selectivity index (CC_50_/IC_50_). ^d^ M: Miltefosine was used as a positive control against *L. amazonensis*. ^e^ B: Benznidazole was used as the positive controls against *T. cruzi*. Antikinetoplastid activity and cytotoxicity assays were performed as independent experiments in triplicates. Derivatives **2** and **6** were excluded due to their low antiparasitic activity.

**Table 2:** Kinetoplastid activity of derivatives (**9** and **10**) against intramacrophage of *L. amazonensis* and *T. cruzi*, and cytotoxicity against murine macrophage cells.

Cmpds	*L. amazonensis* Intracellular	*T. cruzi* Intracellular	Cytotoxicity	Selectivity *Leishmania*	Selectivity *Trypanosoma*
	IC_50_^a^	IC_50_^a^	CC_50_^b^	SI ^c^	SI ^d^
**9**	15.2 ± 0.8	9.1 ± 0.5	>300	>19.7	>33.0
**10**	25.3 ± 0.8	28.8 ± 5.6	254.5 ± 1.9	10.1	8.8
M ^d^	3.1 ± 0.3		72.2 ± 8.9	23.3	
B ^e^		2.7 ± 0.4	399.9 ± 1.4		148.1

^a^ IC_50_: Concentrations able to inhibit 50% of parasites expressed as µM ± standard deviation (SD). ^b^ CC_50_: Concentration able to inhibit 50% of murine macrophages expressed as µM ± standard deviation (SD). ^c^ SI: Selectivity index (CC_50_/IC_50_). ^d^ M: Miltefosine was used as a positive control against *L. amazonensis*. ^e^ B: Benznidazole was used as the positive controls against *T. cruzi*. Antikinetoplastid activity and cytotoxicity assays were performed as independent experiments in triplicates.

**Table 3:** Predicted physicochemical parameters according to Lipinski’s rule and drug-likeness for derivatives **9** and **10**.

Cmpds	Parameters of Lipinski’s Rule							Drug-Likeness
	Log P	TPSA	MW	HBA	HBD	RB	Vs	
**9**	4.25	35.53	284.35	3	0	5	0	Yes
**10**	3.48	39.19	255.31	3	0	4	0	Yes

Log *P*: Octanol/water partition coefficient. TPSA: Topological polar surface area. MW: Molecular weight. HBA: Number of hydrogen bond acceptors. HBD: Number of hydrogen bond donors. RB: Number of rotatable bonds. Vs: Number of violations from Lipinski’s rule.

**Table 4:** Bioavailability—pharmacokinetic and metabolic properties of derivatives **9** and **10**.

Properties	Derivatives		Properties	Derivatives	
	9	10		9	10
BA	0.55	0.55	CYP1A2	Yes	Yes
GI	High	High	CYP2C19	Yes	Yes
BBB	Yes	Yes	CYP2C9	No	Yes
P-gp	No	No	CYP2D6	Yes	Yes
Log *Kp*	−4.63	−5.51	CYP3A4	No	No

BA: Bioavaibility. GI: Gastrointestinal absorption. BBB: Blood–brain barrier permeation. P-gp: P-glycoprotein substrate. Log *Kp*: Skin permeation. Cytochrome isoenzymes: CYP1A2, CYP2C19, CYP2C9, CYP2D6, and CYP3A4.

## Data Availability

The original contributions presented in this study are included in the article/Supplementary Material. Further inquiries can be directed to the corresponding author.

## Supplementary Materials

The following supporting information can be downloaded at: https://www.mdpi.com/article/10.3390/antibiotics14040383/s1. Spectroscopic Data, ADME Profiles, and IC50/CC50 Measurements.
