# Supplementary material for: Mechanism Insight of Cell Death Signaling by Thymol Derivatives on Trypanosomatidae Protozoan Parasites
Source: Antibiotics (Basel). 2025 Apr 5;14(4):383. doi: 10.3390/antibiotics14040383 (PMC12024213; doi:10.3390/antibiotics14040383)
Supplement: Supplementary file 1 [file antibiotics-14-00383-s001.zip › antibiotics-3522749-supplementary.pdf]

Mechanism Insight of Cell Death Signaling by Thymol Derivatives on Trypanosmatidae Protozoan Parasites

Amani Omrani[1,2,3#], Meriam Ben Youssef[1,2,3#], Ines Sifaoui[1,4,5], Eduardo Hernández-Álvarez[3], Carlos J. Bethencourt-Estrella[1,4,5], Isabel L. Bazzocchi[3], Hichem Sebai[2], Jacob Lorenzo-Morales[1,4,5], Ignacio A. Jiménez[3,*], José E. Piñero[1,4,5]

[1]Instituto Universitario de Enfermedades Tropicales y Salud Pública de Canarias, Universidad de La Laguna, 38296 La Laguna, Tenerife, Spain

[2]Laboratory of Functional Physiology and Valorization of Bio-Ressources, Higher Institute of Biotechnology of Beja, University of Jendouba, Beja 382-9000, Tunisia

[3]Instituto Universitario de Bio-Orgánica Antonio González, and Departamento de Química Orgánica, Universidad de La Laguna, Avenida Astrofísico Francisco Sánchez 2, 38206 La Laguna, Tenerife, Spain

[4]Departamento de Obstetricia y Ginecología, Pediatría, Medicina Preventiva y Salud Pública, Toxicología, Medicina Legal y Forense y Parasitología, Universidad de La Laguna, C/ Sta. María Soledad s/n, 38200 La Laguna, Tenerife, Spain

[5]Consorcio Centro de Investigación Biomédica en Red, Área de Enfermedades Infecciosas, Instituto de Salud (CIBERINFEC) Carlos III, Av. Monforte de Lemos 3-5, Pabellón 11, 28029 Madrid, Spain

[#] Both authors contributed equally to this work.

* Corresponding author: ignadiaz@ull.edu.es

Electronic Supporting Information

Table of contents

Page 3: **Figures S1 and S2**, ^{1}H NMR and ^{13}C NMR spectra of compound **1**

Page 4: **Figures S3, S4 and S5**, ^{1}H NMR, ^{13}C NMR and Mass spectra of compound **2**

Page 5: **Figures S6, S7 and S8**, ^{1}H NMR, ^{13}C NMR and Mass spectra of compound **3**

Page 6: **Figures S9, S10 and S11**, ^{1}H NMR, ^{13}C NMR and Mass spectra of compound **4**

Page 7: **Figures S12, S13 and S14**, ^{1}H NMR, ^{13}C NMR and Mass spectra of compound **5**

Page 8: **Figures S15, S16 and S17**, ^{1}H NMR, ^{13}C NMR and Mass spectra of compound **6**

Page 9: **Figures S18, S19 and S20**, ^{1}H NMR, ^{13}C NMR and Mass spectra of compound **7**

Page 10: **Figures S21, S22 and S23**, ^{1}H NMR, ^{13}C NMR and Mass spectra of compound **8**

Page 11: **Figures S24, S25 and S26**, ^{1}H NMR, ^{13}C NMR and Mass spectra of compound **9**

Page 12: **Figures S27, S28 and S29**, ^{1}H NMR and ^{13}C NMR and Mass spectra of compound **10**

Page 13: **Figures S30, S31 and S32**, ^{1}H NMR, ^{13}C NMR and Mass spectra of compound **11**

Page 14: **Figures S33, S34 and S35**, ^{1}H NMR, ^{13}C NMR and Mass spectra of compound **12**

Page 15: **Figure S36 and S37,** ADME prediction of compounds **1 and 2**

Page 16: **Figure S38 and S39,** ADME prediction of compounds **3 and 4**

Page 17: **Figure S40 and S41,** ADME prediction of compounds **5 and 6**

Page 18: **Figure S42 and S43,** ADME prediction of compounds **7 and 8**

Page 19: **Figure S44 and S45,** ADME prediction of compound **9 and 10**

Page 20: **Figure S46 and S47,** ADME prediction of compound **11 and 12**

Page 21: **Figure S48,** IC_{50} measurements across replicates of *Leishmania amazonensis*

Page 21: **Figure S49,** IC_{50} measurements across replicates of *Trypanosoma cruzi*

Page 22: **Figure S50**, curves for CC_{50} measurements

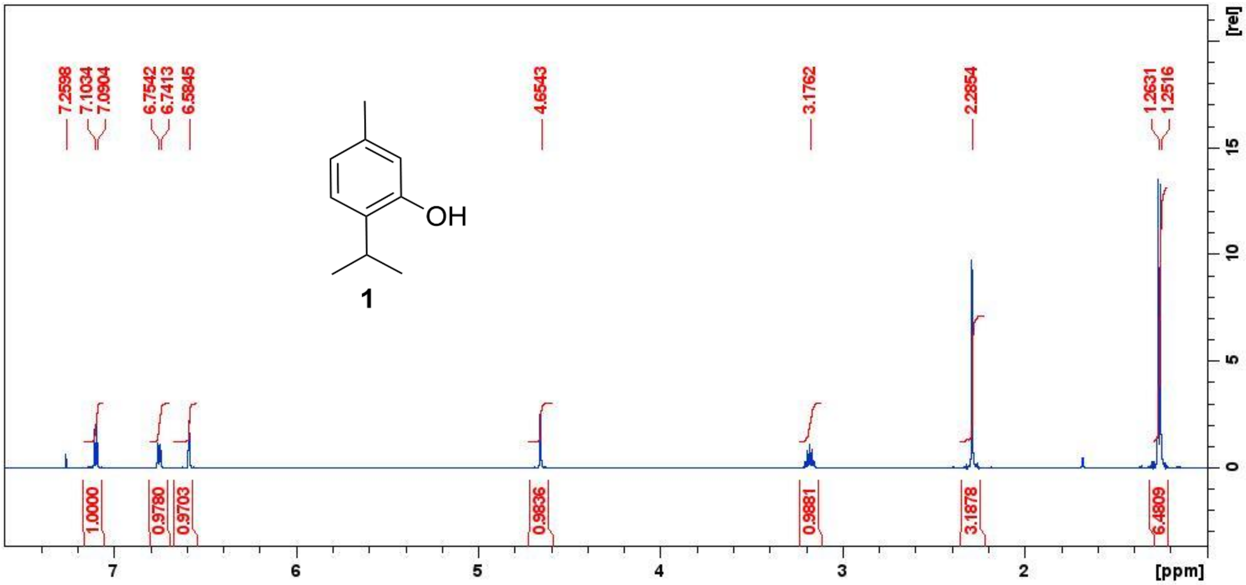

Figure S1. ^{1}H NMR spectrum [600 MHz, solvent $CDCl_3$] of compound **1**.

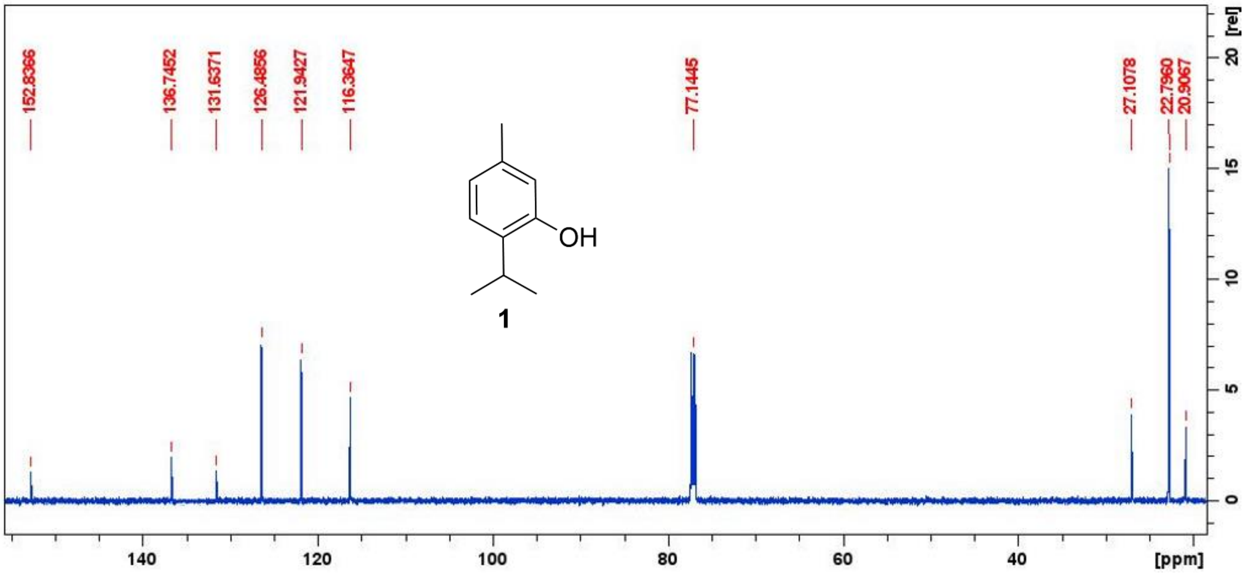

Figure S2. ^{13}C NMR spectrum [150 MHz, solvent $CDCl_3$] of compound **1**.

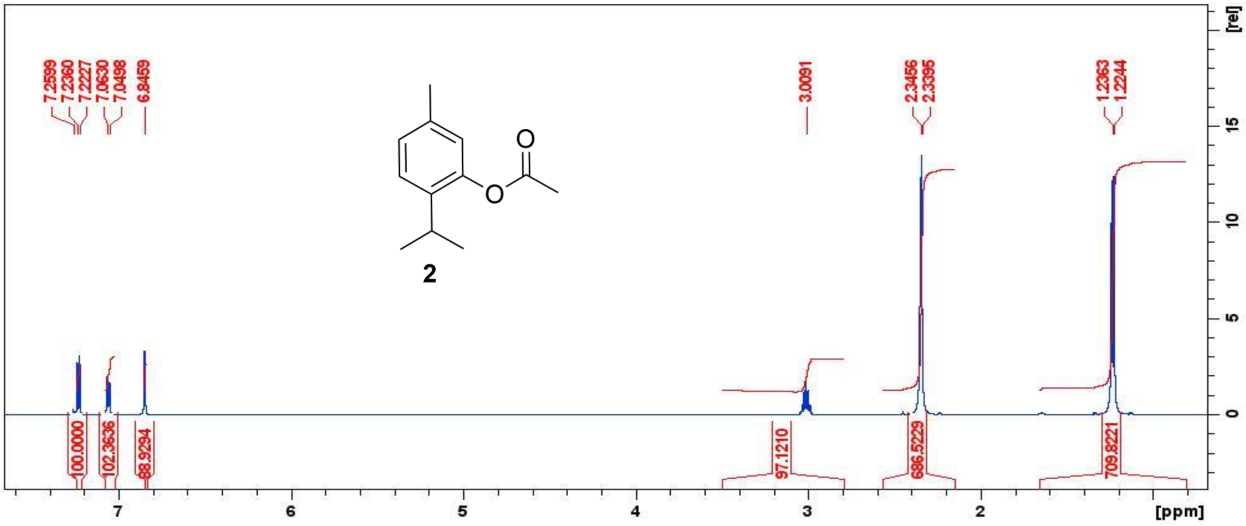

Figure S3. ^{1}H NMR spectrum [600 MHz, solvent $CDCl_3$] of compound **2**.

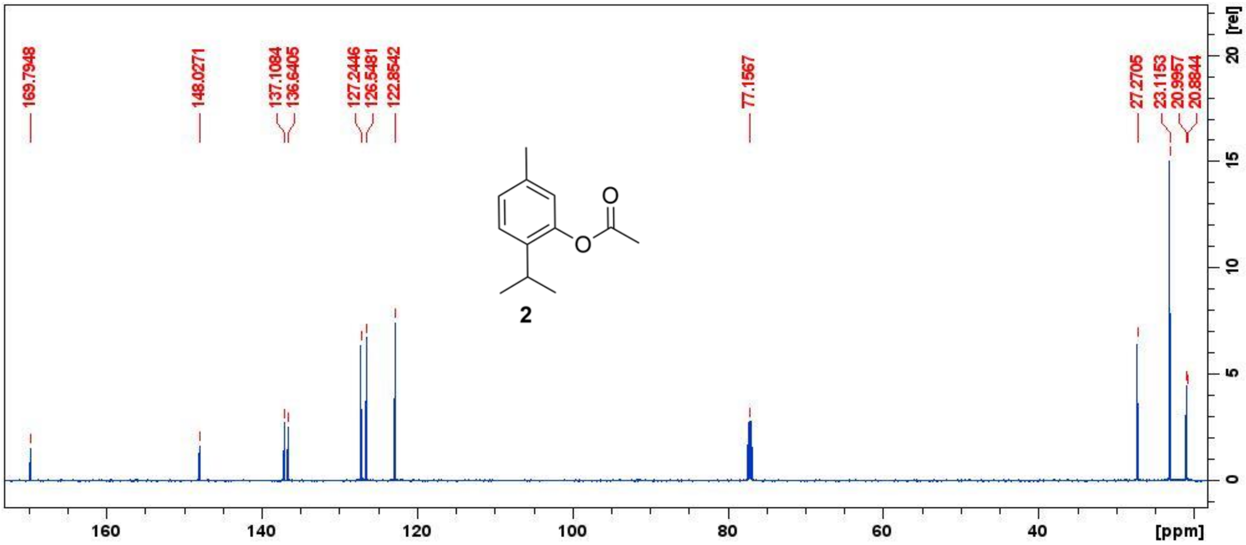

Figure S4. ^{13}C NMR spectrum [150 MHz, solvent $CDCl_3$] of compound **2**.

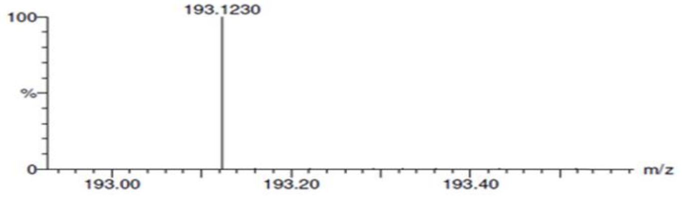

Minimum:	40.00				-1.5			
Maximum:	100.00		5.0	5.0	200.0			
Mass	RA	Calc. Mass	mDa	PPM	DBE	i-FIT	i-FIT (Norm)	Formula
193.1230	100.00	193.1229	0.1	0.5	4.5	75.2	0.0	C12 H17 O2

Figure S5. Mass spectrum of compound **2** (molecular ion $[M+H]^{+}$).

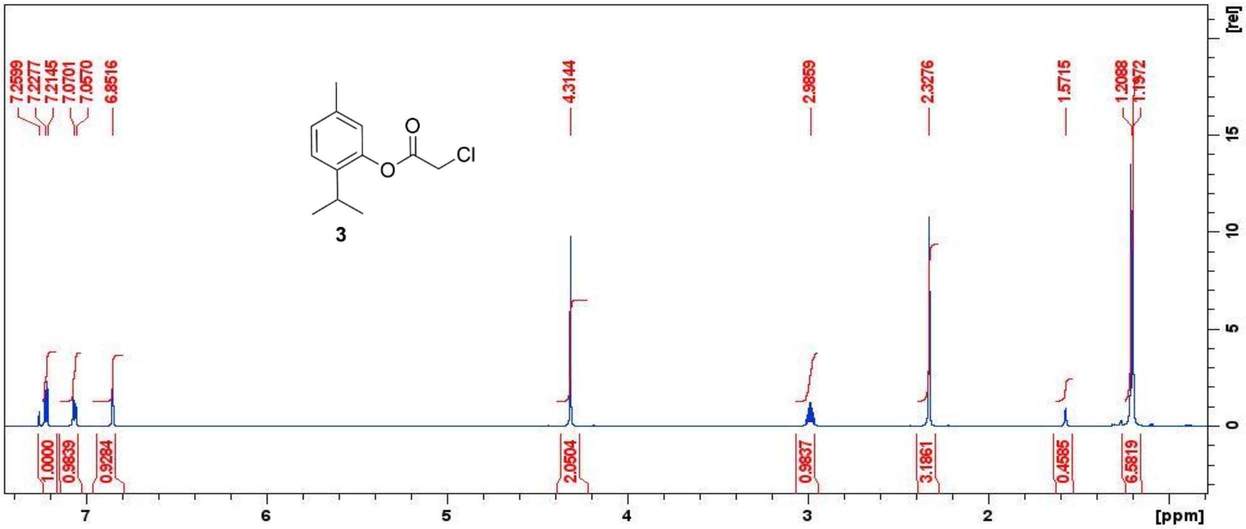

Figure S6. ^{1}H NMR spectrum [600 MHz, solvent $CDCl_3$] of compound **3**.

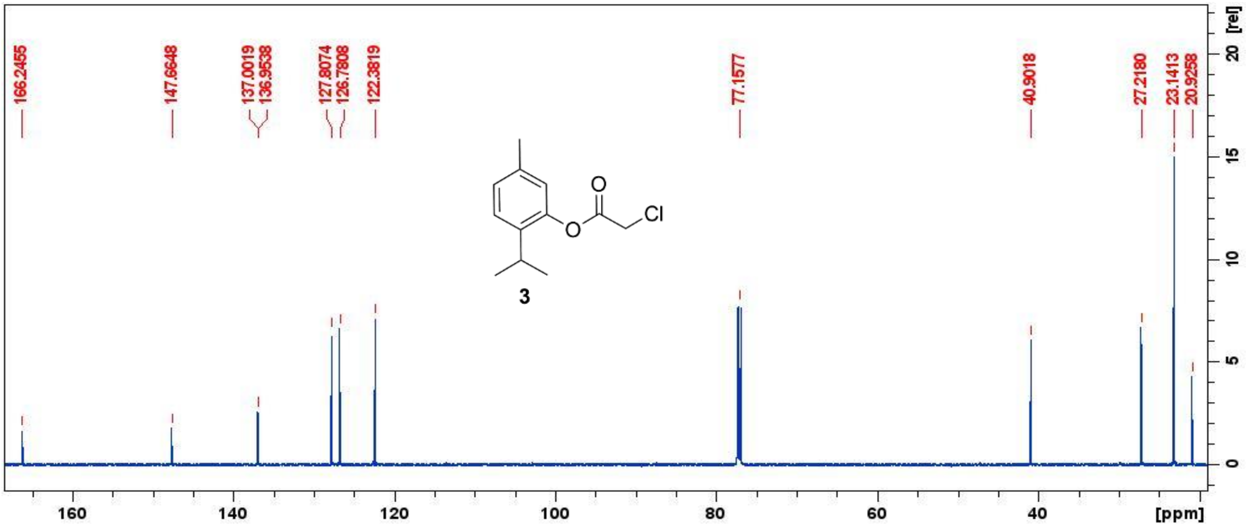

Figure S7. ^{13}C NMR spectrum [150 MHz, solvent $CDCl_3$] of compound **3**.

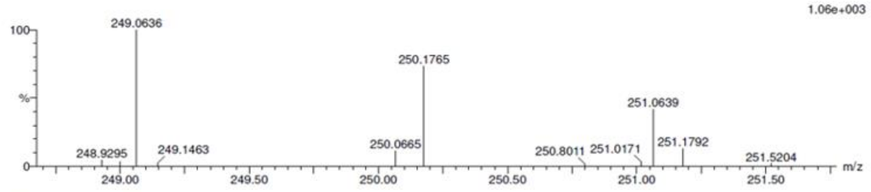

Minimum:	40.00				-1.5			
Maximum:	100.00		5.0	5.0	200.0			
Mass	RA	Calc. Mass	mDa	PPM	DBE	i-FIT	i-FIT (Norm)	Formula
251.0639	41.61	251.0629	1.0	4.0	4.5	54.4	0.7	C12 H15 O2 Na 37Cl

Figure S8. Mass Spectrum of compound **3** (molecular ion $[M+Na]^+$).

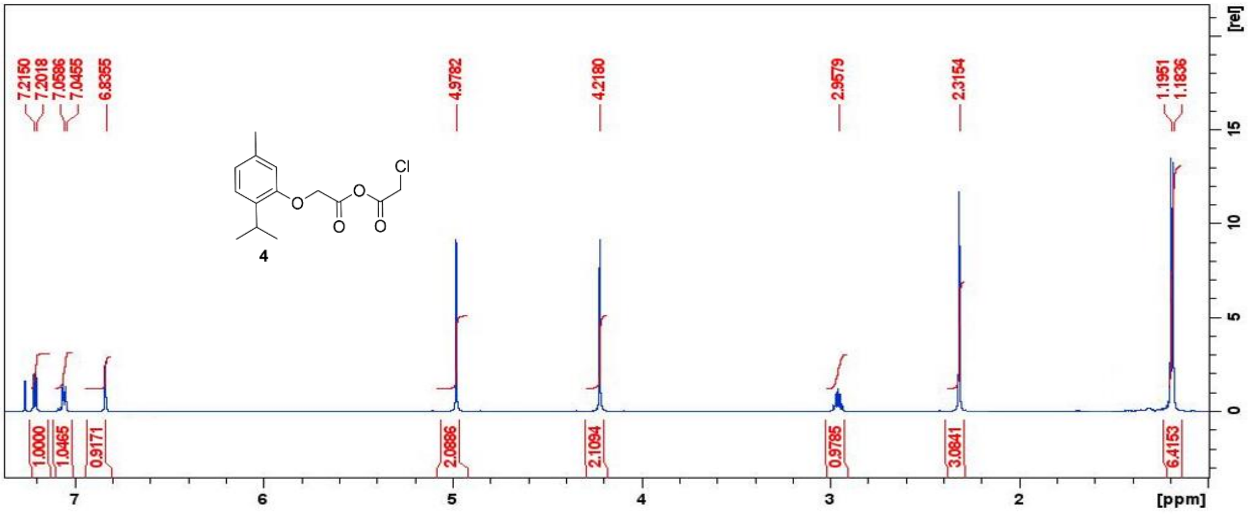

Figure S9. ^{1}H NMR spectrum [600 MHz, solvent $CDCl_3$] of compound **4**.

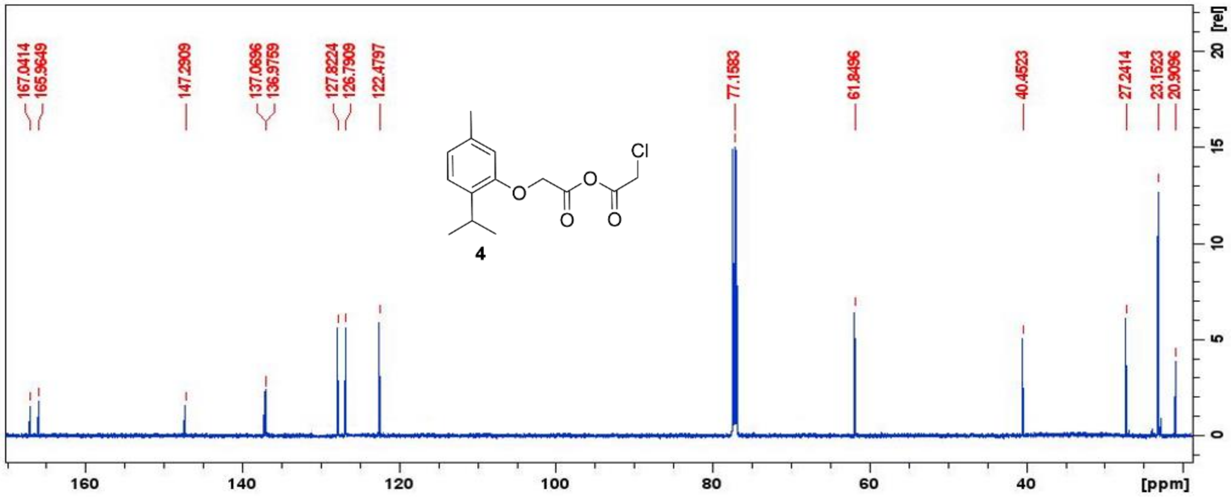

Figure S10. ^{13}C NMR spectrum [150 MHz, solvent $CDCl_3$] of compound **4**.

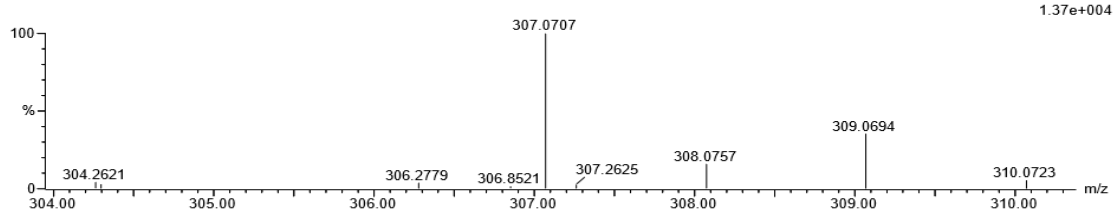

Minimum:	20.00				-1.5			
Maximum:	100.00		5.0	5.0	200.0			
Mass	RA	Calc. Mass	mDa	PPM	DBE	i-FIT	i-FIT (Norm)	Formula
307.0707	100.00	307.0713	-0.6	-2.0	5.5	41.9	1.9	C14 H17 O4 Na 35Cl
309.0694	35.35	309.0684	1.0	3.2	5.5	23.3	3.3	C14 H17 O4 Na 37Cl

Figure S11. Mass Spectrum of compound **4** (molecular ion $[M+Na]^+$).

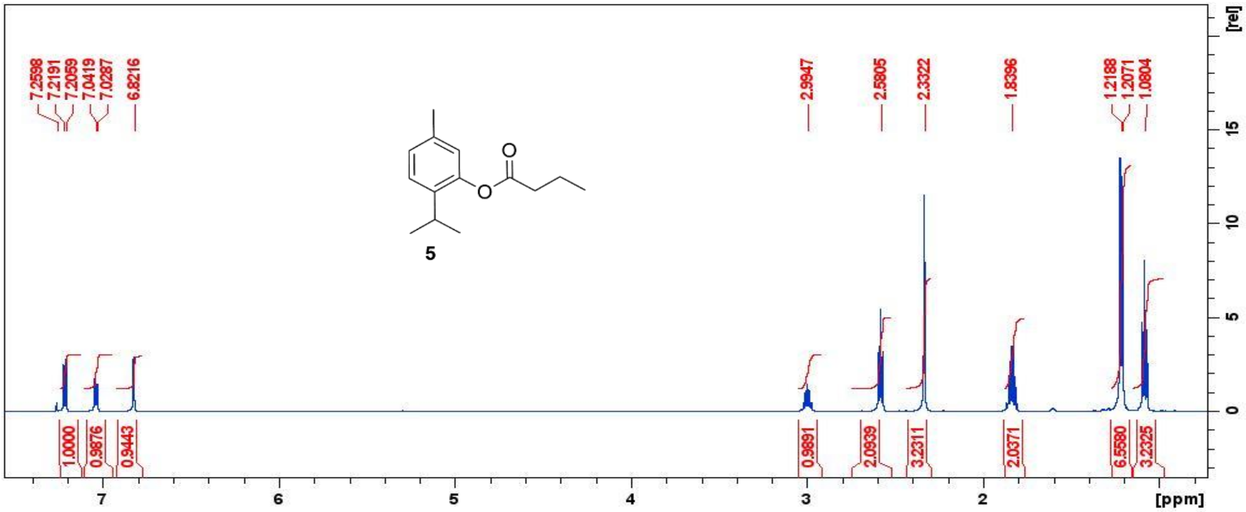

Figure S12. ^{1}H NMR spectrum [600 MHz, solvent $CDCl_3$] of compound **5**.

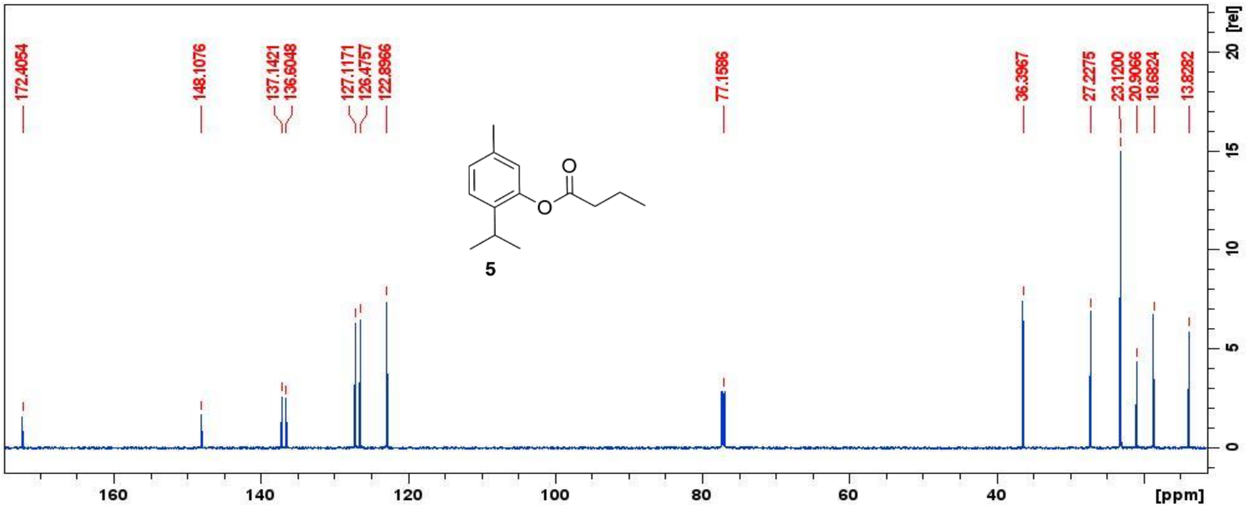

Figure S13. ^{13}C NMR spectrum [150 MHz, solvent $CDCl_3$] of compound **5**

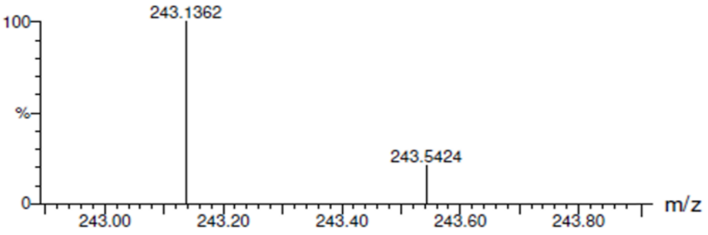

Minimum:	40.00				-1.5			
Maximum:	100.00		5.0	5.0	200.0			
Mass	RA	Calc. Mass	mDa	PPM	DBE	i-FIT	i-FIT (Norm)	Formula
243.1362	100.00	243.1361	0.1	0.4	4.5	84.8	0.0	C14 H20 O2 Na

Figure S14. Mass Spectrum of compound **5** (molecular ion $[M+Na]^{+}$).

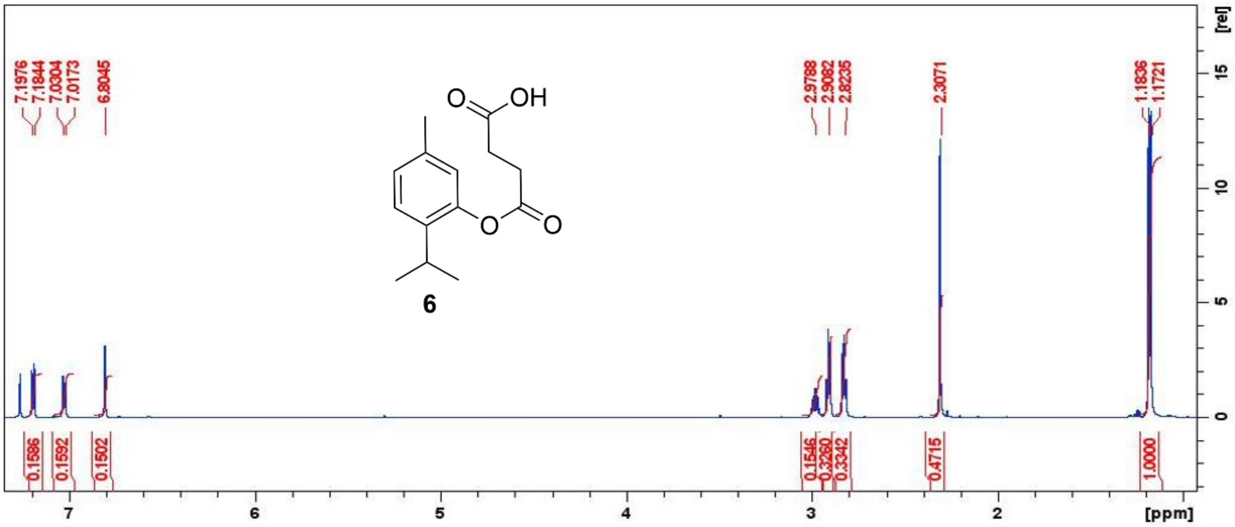

Figure S15. ^{1}H NMR spectrum [600 MHz, solvent $CDCl_3$] of compound **6**.

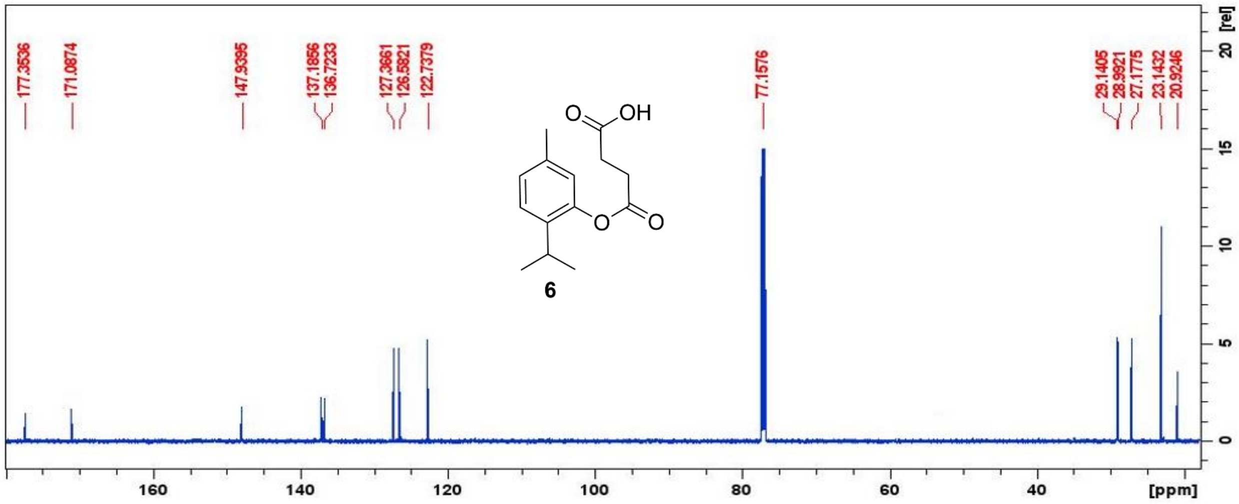

Figure S16. ^{13}C NMR spectrum [150 MHz, solvent $CDCl_3$] of compound **6**.

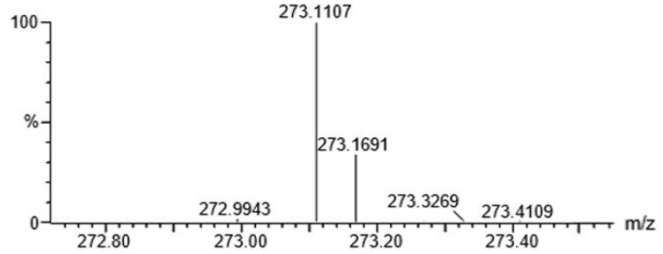

Minimum:	20.00				-1.5			
Maximum:	100.00		5.0	5.0	200.0			
Mass	RA	Calc. Mass	mDa	PPM	DBE	i-FIT	i-FIT (Norm)	Formula
273.1107	100.00	273.1103	0.4	1.5	5.5	116.2	0.0	C14 H18 O4 Na

Figure S17. Mass Spectrum of compound **6** (molecular ion [M+Na]$^+$).

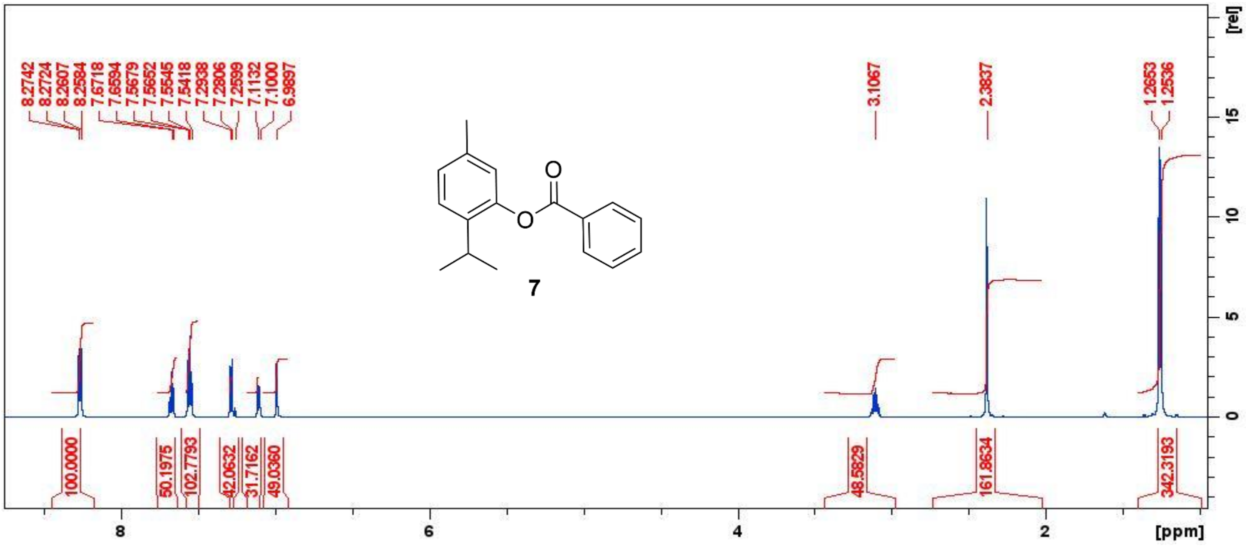

Figure S18. ^{1}H NMR spectrum [600 MHz, solvent $CDCl_3$] of compound **7**.

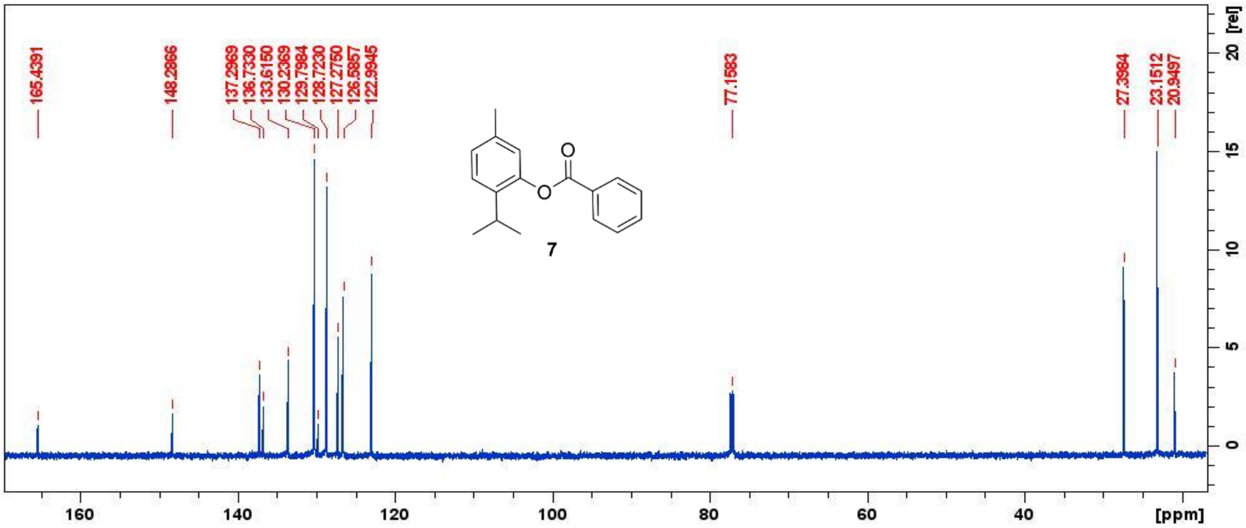

Figure S19. ^{13}C NMR spectrum [150 MHz, solvent $CDCl_3$] of compound **7**.

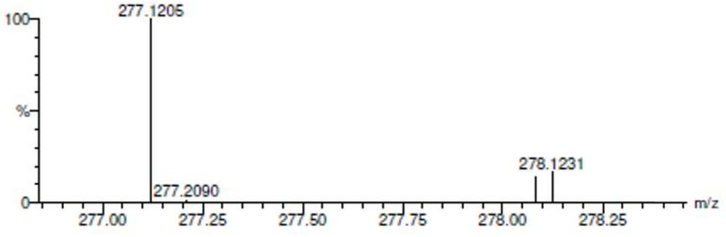

Minimum:	40.00				-1.5			
Maximum:	100.00		5.0	5.0	200.0			
Mass	RA	Calc. Mass	mDa	PPM	DBE	i-FIT	i-FIT (Norm)	Formula
277.1205	100.00	277.1204	0.1	0.4	8.5	52.7	0.0	C17 H18 O2 Na

Figure S20. Mass Spectrum of compound **7** (molecular ion $[M+Na]^{+}$).

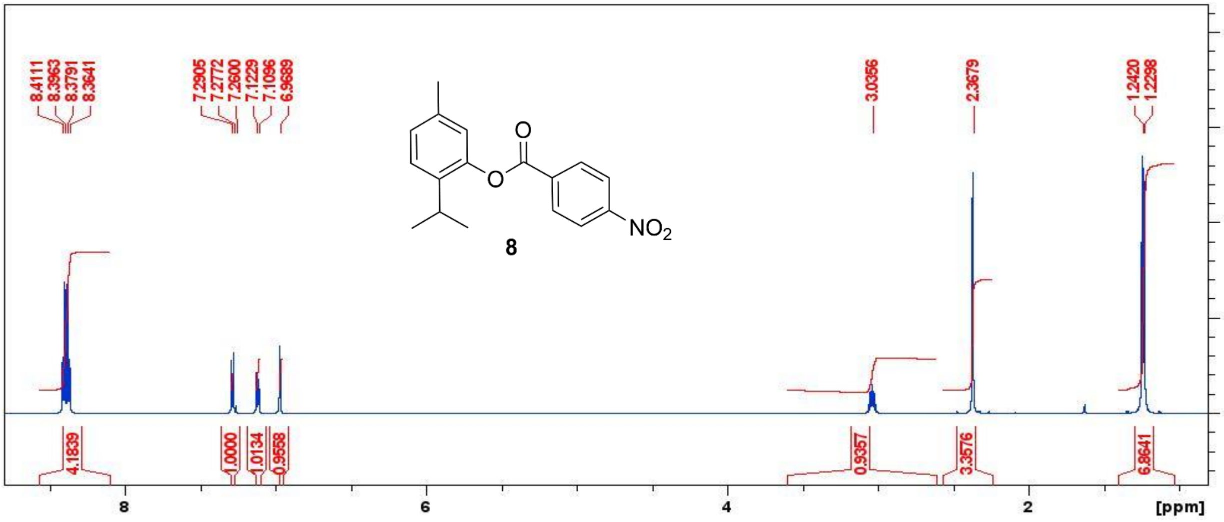

Figure S21. ^{1}H NMR spectrum [600 MHz, solvent $CDCl_3$] of compound **8**.

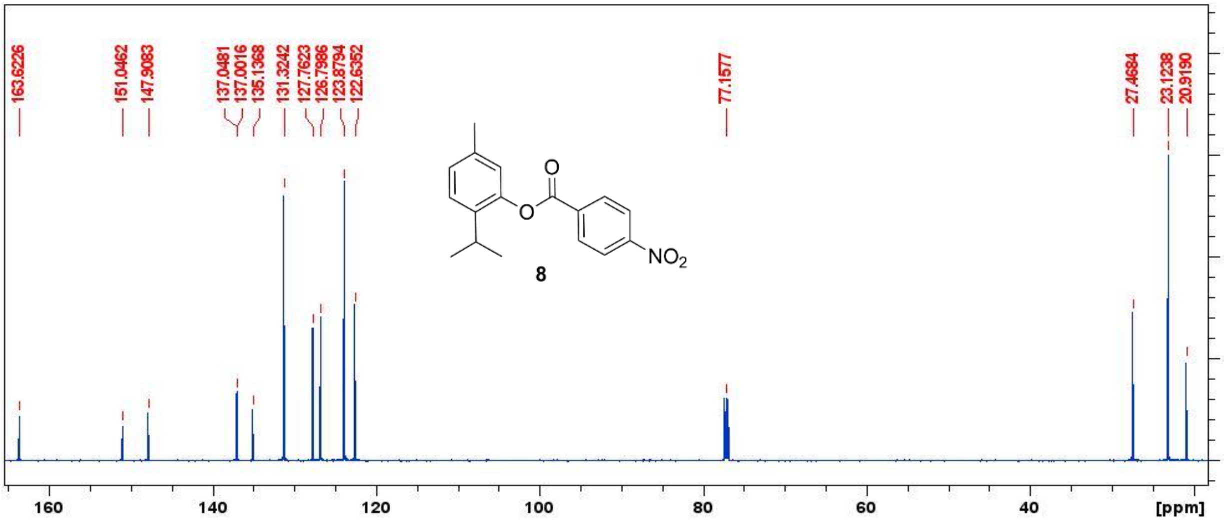

Figure S22. ^{13}C NMR spectrum [150 MHz, solvent $CDCl_3$] of compound **8**.

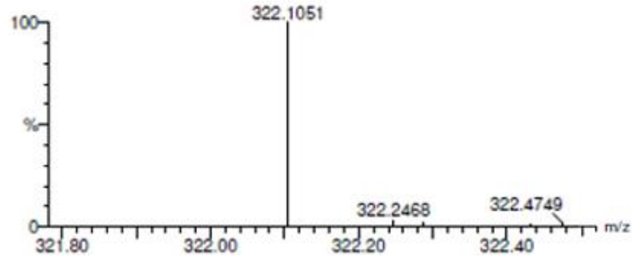

Minimum:	40.00				-1.5			
Maximum:	100.00		5.0	5.0	200.0			
Mass	RA	Calc. Mass	mDa	PPM	DBE	i-FIT	i-FIT (Norm)	Formula
322.1051	100.00	322.1055	-0.4	-1.2	9.5	81.7	0.0	C17 H17 N O4 Na

Figure S23. Mass Spectrum of compound **8** (molecular ion $[M+Na]^{+}$).

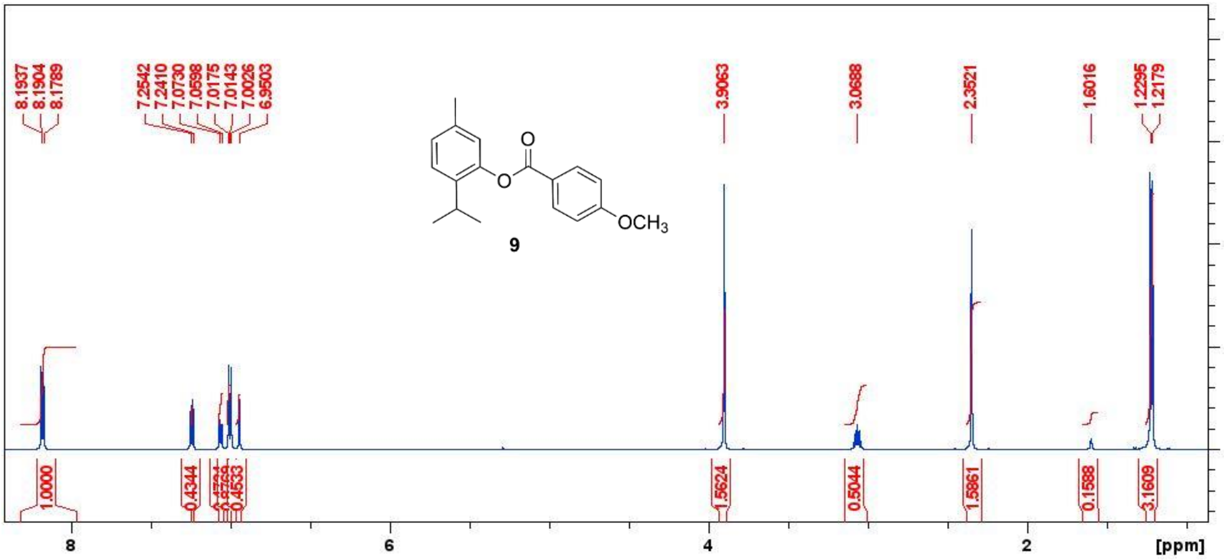

Figure S24. ^{1}H NMR spectrum [600 MHz, solvent $CDCl_3$] of compound **9**.

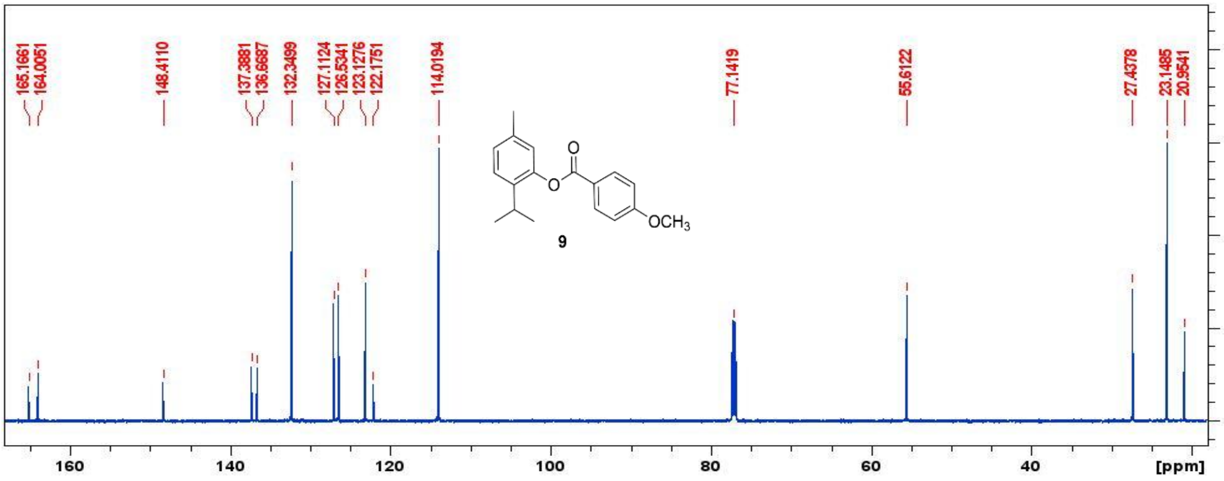

Figure S25. ^{13}C NMR spectrum [150 MHz, solvent $CDCl_3$] of compound **9**.

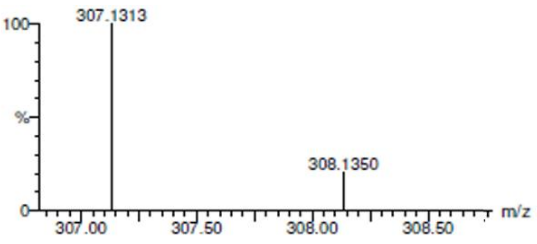

Mass	RA	Calc. Mass	mDa	PPM	DBE	i-FIT	i-FIT (Norm)	Formula
Minimum:	5.00				-1.5			
Maximum:	100.00		5.0	5.0	200.0			
307.1313	100.00	307.1310	0.3	1.0	8.5	20.6	0.0	C18 H20 O3 Na

Figure S26. Mass Spectrum of compound **9** (molecular ion [M+Na]$^{+}$).

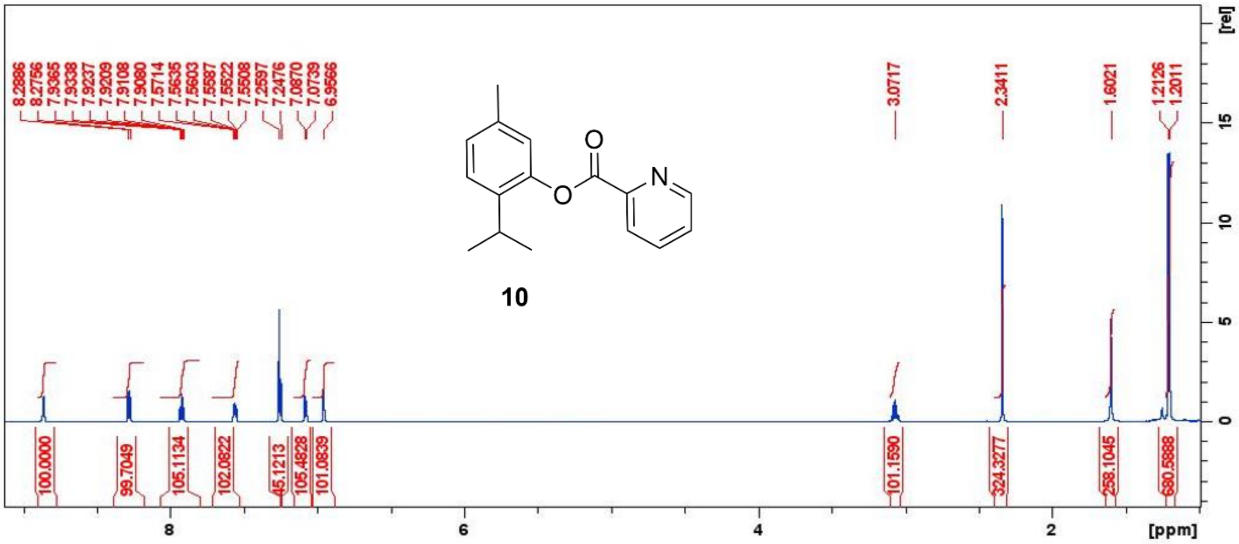

Figure S27. ^{1}H NMR spectrum [600 MHz, solvent $CDCl_3$] of compound **10**.

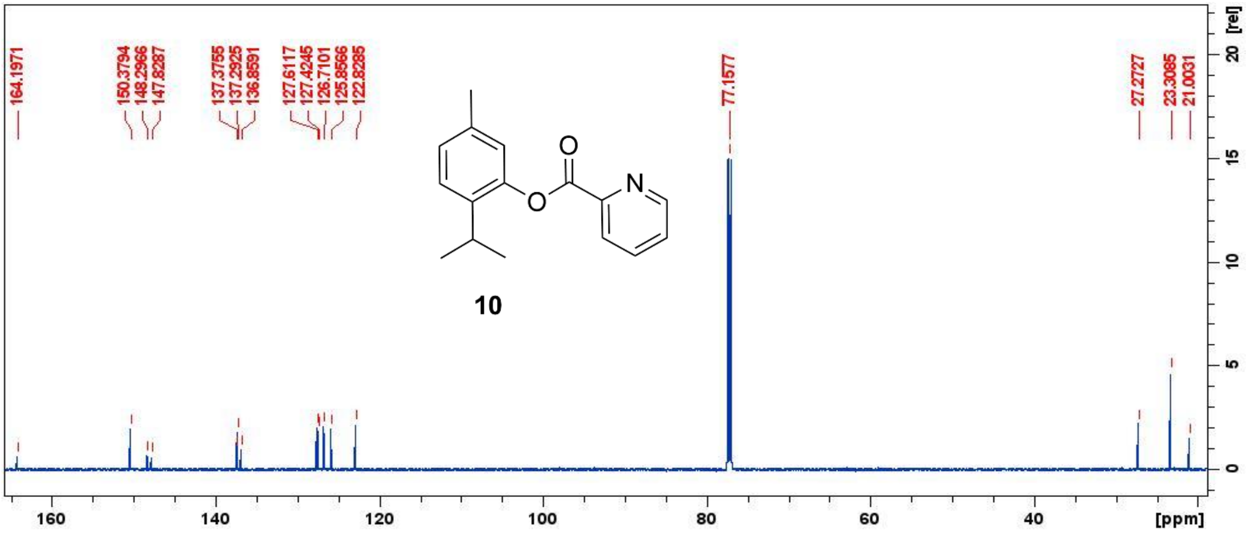

Figure S28. ^{13}C NMR spectrum [150 MHz, solvent $CDCl_3$] of compound **10**.

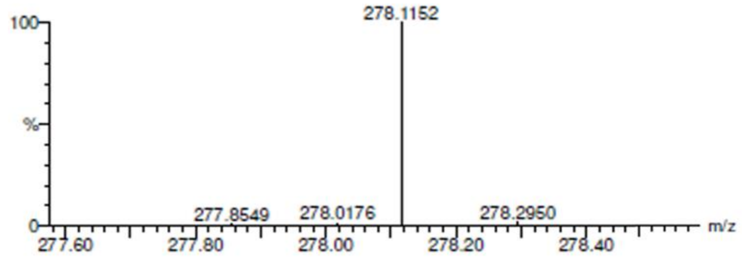

Minimum:	50.00				-1.5			
Maximum:	100.00		5.0	5.0	200.0			
Mass	RA	Calc. Mass	mDa	PPM	DBE	i-FIT	i-FIT (Norm)	Formula
278.1152	100.00	278.1157	-0.5	-1.8	8.5	52.3	0.2	C16 H17 N O2 Na

Figure S29. Mass Spectrum of compound **10** (molecular ion $[M+Na]^{+}$).

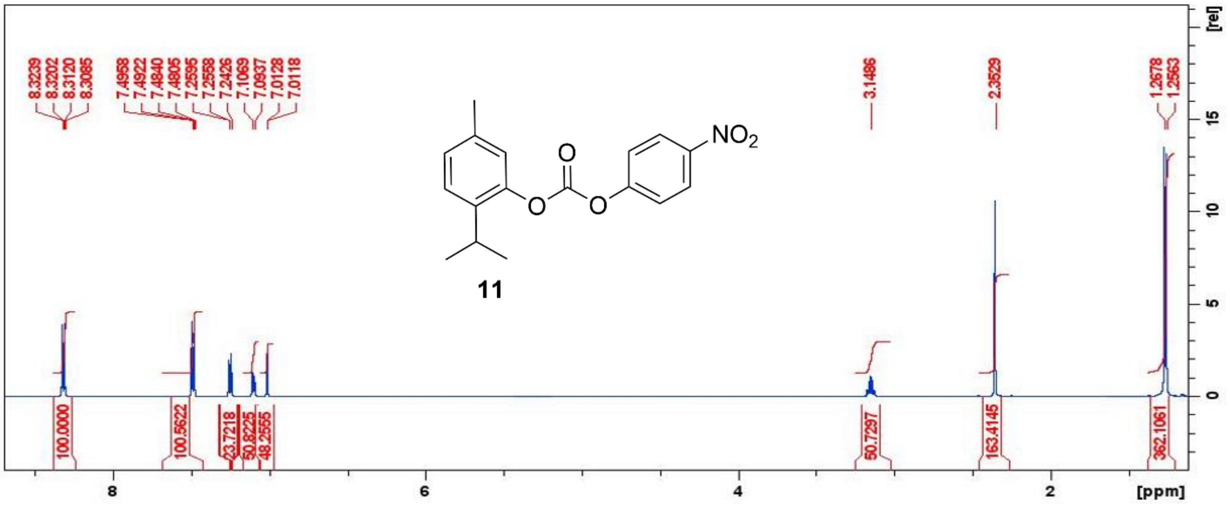

Figure S30. ^{1}H NMR spectrum [600 MHz, solvent $CDCl_3$] of compound **11**.

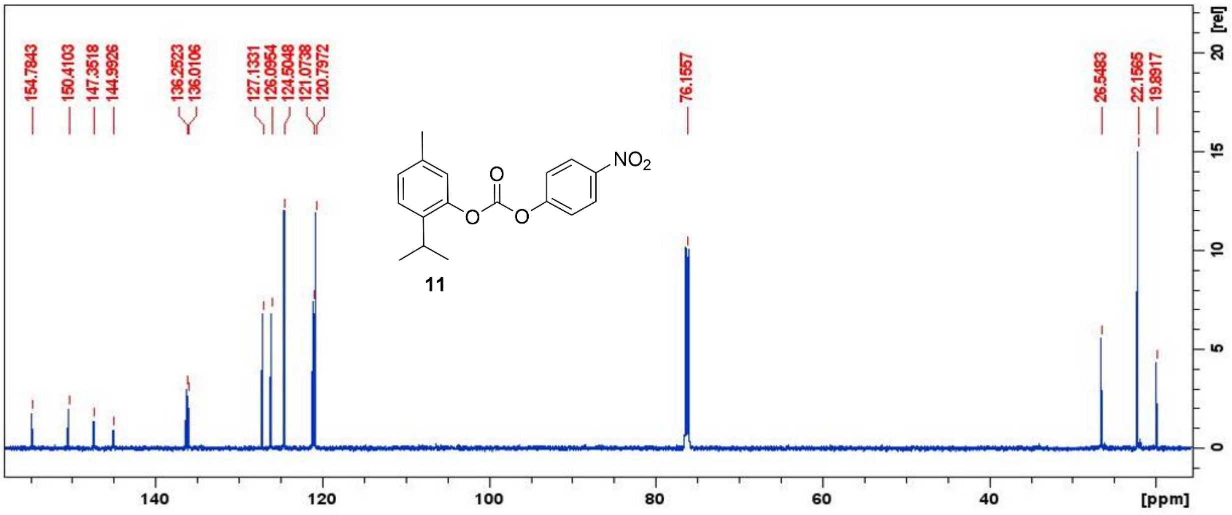

Figure S31. ^{13}C NMR spectrum [150 MHz, solvent $CDCl_3$] of compound **11**.

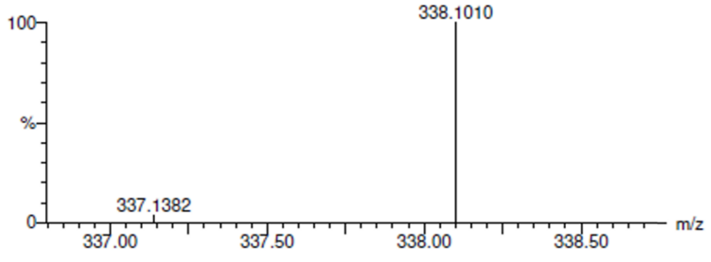

Minimum:	20.00				-1.5			
Maximum:	100.00		5.0	5.0	200.0			
Mass	RA	Calc. Mass	mDa	PPM	DBE	i-FIT	i-FIT (Norm)	Formula
338.1010	100.00	338.1004	0.6	1.8	9.5	44.6	0.0	C17 H17 N O5 Na

Figure S32. Mass spectrum of compound **11** (molecular ion $[M+Na]^{+}$).

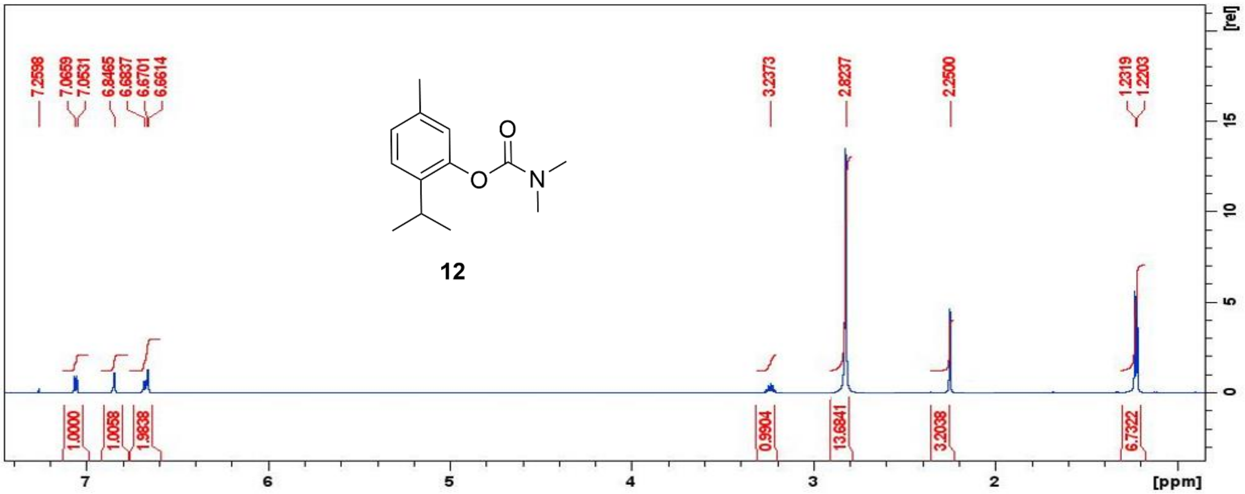

Figure S33. ^{1}H NMR spectrum [600 MHz, solvent $CDCl_3$] of compound **12**.

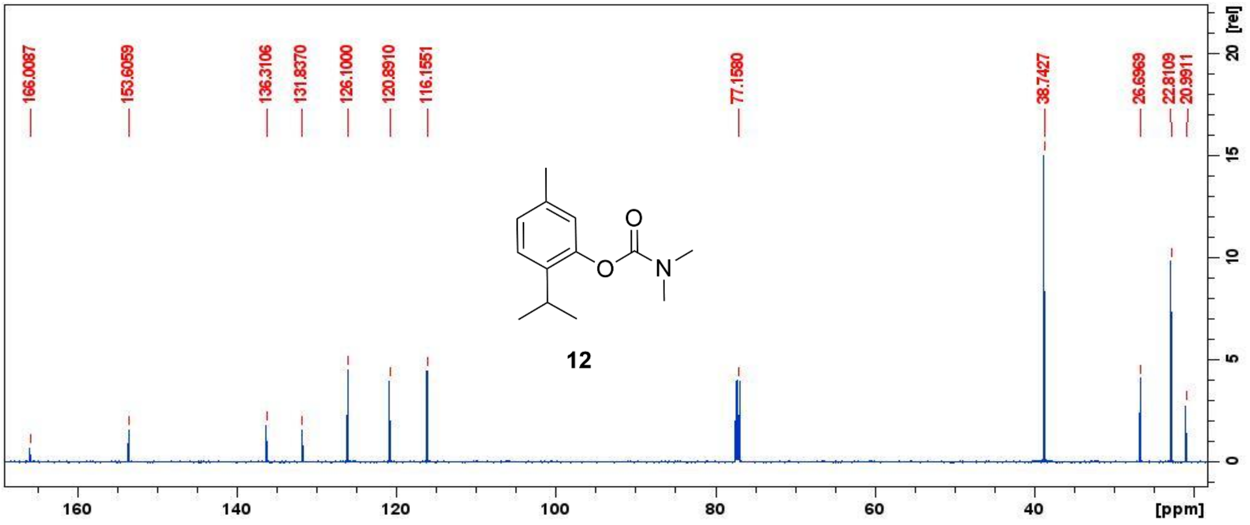

Figure S34. ^{13}C NMR spectrum [150 MHz, solvent $CDCl_3$] of compound **12**.

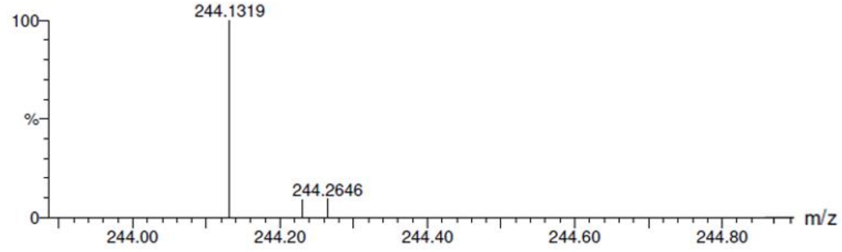

	Mass	RA	Calc. Mass	mDa	PPM	DBE	i-FIT	i-FIT (Norm)	Formula
Minimum:		20.00				-1.5			
Maximum:		100.00		5.0	5.0	200.0			
	244.1319	100.00	244.1313	0.6	2.5	4.5	51.0	0.0	C13 H19 N O2 Na

Figure S35. Mass Spectrum of compound **12** (molecular ion $[M+Na]^{+}$).

Molecule 1

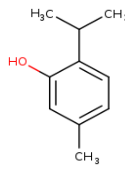

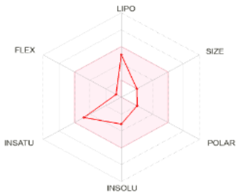

SMILES Cc1ccc(c(c1)O)C(C)C

Physicochemical Properties	
Formula	C10H14O
Molecular weight	150.22 g/mol
Num. heavy atoms	11
Num. arom. heavy atoms	6
Fraction Csp3	0.40
Num. rotatable bonds	1
Num. H-bond acceptors	1
Num. H-bond donors	1
Molar Refractivity	48.01
TPSA	20.23 Å²
**Lipophilicity**	
Log $P_{o/w}$ (iLOGP)	2.32
Log $P_{o/w}$ (XLOGP3)	3.30
Log $P_{o/w}$ (WLOGP)	2.82
Log $P_{o/w}$ (MLOGP)	2.76
Log $P_{o/w}$ (SILICOS-IT)	2.79
Consensus Log $P_{o/w}$	2.80

Water Solubility	
Log *S* (ESOL)	-3.19
Solubility	9.74e-02 mg/ml ; 6.49e-04 mol/l
Class	Soluble
Log *S* (Ali)	-3.40
Solubility	5.97e-02 mg/ml ; 3.98e-04 mol/l
Class	Soluble
Log *S* (SILICOS-IT)	-3.01
Solubility	1.46e-01 mg/ml ; 9.71e-04 mol/l
Class	Soluble
**Pharmacokinetics**	
GI absorption	High
BBB permeant	Yes
P-gp substrate	No
CYP1A2 inhibitor	Yes
CYP2C19 inhibitor	No
CYP2C9 inhibitor	No
CYP2D6 inhibitor	No
CYP3A4 inhibitor	No
Log K_p (skin permeation)	-4.87 cm/s
**Druglikeness**	
Lipinski	Yes; 0 violation
Ghose	No; 1 violation: MW<160
Veber	Yes
Egan	Yes
Muegge	No; 2 violations: MW<200, Heteroatoms<2
Bioavailability Score	0.55
**Medicinal Chemistry**	
PAINS	0 alert
Brenk	0 alert
Leadlikeness	No; 1 violation: MW<250
Synthetic accessibility	1.00

Figure S36. Predicted pharmacokinetic parameters of compound **1.**

Molecule 2

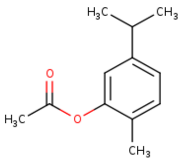

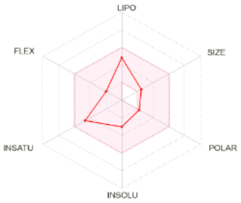

SMILES CC(=O)Oc1cc(ccc1C)C(C)C

Physicochemical Properties	
Formula	C12H16O2
Molecular weight	192.25 g/mol
Num. heavy atoms	14
Num. arom. heavy atoms	6
Fraction Csp3	0.42
Num. rotatable bonds	3
Num. H-bond acceptors	2
Num. H-bond donors	0
Molar Refractivity	57.49
TPSA	26.30 Å²
**Lipophilicity**	
Log $P_{o/w}$ (iLOGP)	2.74
Log $P_{o/w}$ (XLOGP3)	2.96
Log $P_{o/w}$ (WLOGP)	3.04
Log $P_{o/w}$ (MLOGP)	3.13
Log $P_{o/w}$ (SILICOS-IT)	3.21
Consensus Log $P_{o/w}$	3.02

Water Solubility	
Log *S* (ESOL)	-3.02
Solubility	1.85e-01 mg/ml ; 9.64e-04 mol/l
Class	Soluble
Log *S* (Ali)	-3.18
Solubility	1.28e-01 mg/ml ; 6.68e-04 mol/l
Class	Soluble
Log *S* (SILICOS-IT)	-3.69
Solubility	3.95e-02 mg/ml ; 2.06e-04 mol/l
Class	Soluble
**Pharmacokinetics**	
GI absorption	High
BBB permeant	Yes
P-gp substrate	No
CYP1A2 inhibitor	No
CYP2C19 inhibitor	No
CYP2C9 inhibitor	No
CYP2D6 inhibitor	No
CYP3A4 inhibitor	No
Log K_p (skin permeation)	-5.37 cm/s
**Druglikeness**	
Lipinski	Yes; 0 violation
Ghose	Yes
Veber	Yes
Egan	Yes
Muegge	No; 1 violation: MW<200
Bioavailability Score	0.55
**Medicinal Chemistry**	
PAINS	0 alert
Brenk	1 alert: phenol_ester
Leadlikeness	No; 1 violation: MW<250
Synthetic accessibility	1.69

Figure S37. Predicted pharmacokinetic parameters of compound **2.**

Molecule 3

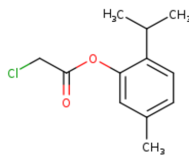

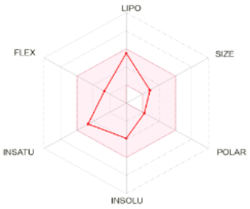

SMILES ClCC(=O)Oc1cc(C)ccc1C(C)C

Physicochemical Properties	
Formula	C12H15ClO2
Molecular weight	226.70 g/mol
Num. heavy atoms	15
Num. arom. heavy atoms	6
Fraction Csp3	0.42
Num. rotatable bonds	4
Num. H-bond acceptors	2
Num. H-bond donors	0
Molar Refractivity	62.28
TPSA	26.30 Å²

Lipophilicity	
Log $P_{o/w}$ (iLOGP)	2.70
Log $P_{o/w}$ (XLOGP3)	4.15
Log $P_{o/w}$ (WLOGP)	3.26
Log $P_{o/w}$ (MLOGP)	3.40
Log $P_{o/w}$ (SILICOS-IT)	3.74
Consensus Log $P_{o/w}$	3.45

Water Solubility	
Log *S* (ESOL)	-3.89
Solubility	2.91e-02 mg/ml ; 1.28e-04 mol/l
Class	Soluble
Log *S* (Ali)	-4.41
Solubility	8.82e-03 mg/ml ; 3.89e-05 mol/l
Class	Moderately soluble
Log *S* (SILICOS-IT)	-4.33
Solubility	1.07e-02 mg/ml ; 4.73e-05 mol/l
Class	Moderately soluble

Pharmacokinetics	
GI absorption	High
BBB permeant	Yes
P-gp substrate	No
CYP1A2 inhibitor	Yes
CYP2C19 inhibitor	No
CYP2C9 inhibitor	No
CYP2D6 inhibitor	No
CYP3A4 inhibitor	No
Log K_p (skin permeation)	-4.74 cm/s

Druglikeness	
Lipinski	Yes; 0 violation
Ghose	Yes
Veber	Yes
Egan	Yes
Muegge	Yes
Bioavailability Score	0.55

Medicinal Chemistry	
PAINS	0 alert
Brenk	2 alerts: alkyl_halide, phenol_ester
Leadlikeness	No; 2 violations: MW<250, XLOGP3>3.5
Synthetic accessibility	2.11

Figure S38. Predicted pharmacokinetic parameters of compound **3.**

Molecule 4

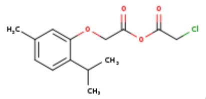

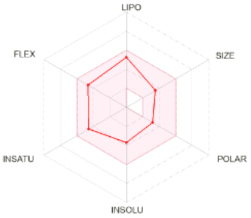

SMILES ClCC(=O)OC(=O)COc1cc(C)ccc1C(C)C

Physicochemical Properties	
Formula	C14H17ClO4
Molecular weight	284.74 g/mol
Num. heavy atoms	19
Num. arom. heavy atoms	6
Fraction Csp3	0.43
Num. rotatable bonds	7
Num. H-bond acceptors	4
Num. H-bond donors	0
Molar Refractivity	73.18
TPSA	52.60 Å²

Lipophilicity	
Log $P_{o/w}$ (iLOGP)	2.36
Log $P_{o/w}$ (XLOGP3)	3.71
Log $P_{o/w}$ (WLOGP)	2.81
Log $P_{o/w}$ (MLOGP)	2.91
Log $P_{o/w}$ (SILICOS-IT)	3.66
Consensus Log $P_{o/w}$	3.09

Water Solubility	
Log *S* (ESOL)	-3.71
Solubility	5.50e-02 mg/ml ; 1.93e-04 mol/l
Class	Soluble
Log *S* (Ali)	-4.51
Solubility	8.89e-03 mg/ml ; 3.12e-05 mol/l
Class	Moderately soluble
Log *S* (SILICOS-IT)	-4.41
Solubility	1.12e-02 mg/ml ; 3.92e-05 mol/l
Class	Moderately soluble

Pharmacokinetics	
GI absorption	High
BBB permeant	Yes
P-gp substrate	No
CYP1A2 inhibitor	Yes
CYP2C19 inhibitor	Yes
CYP2C9 inhibitor	No
CYP2D6 inhibitor	No
CYP3A4 inhibitor	No
Log K_p (skin permeation)	-5.40 cm/s

Druglikeness	
Lipinski	Yes; 0 violation
Ghose	Yes
Veber	Yes
Egan	Yes
Muegge	Yes
Bioavailability Score	0.55

Medicinal Chemistry	
PAINS	0 alert
Brenk	3 alerts: alkyl_halide, beta_keto_anhydride, more_than_2_esters
Leadlikeness	No; 1 violation: XLOGP3>3.5
Synthetic accessibility	2.57

Figure S39. Predicted pharmacokinetic parameters of compound **4.**

Molecule 5

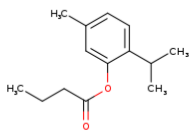

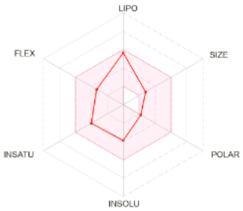

SMILES CCCC(=O)Oc1cc(C)ccc1C(C)C

Physicochemical Properties	
Formula	C14H20O2
Molecular weight	220.31 g/mol
Num. heavy atoms	16
Num. arom. heavy atoms	6
Fraction Csp3	0.50
Num. rotatable bonds	5
Num. H-bond acceptors	2
Num. H-bond donors	0
Molar Refractivity	67.10
TPSA	26.30 Å²

Lipophilicity	
Log $P_{o/w}$ (iLOGP)	3.07
Log $P_{o/w}$ (XLOGP3)	4.36
Log $P_{o/w}$ (WLOGP)	3.82
Log $P_{o/w}$ (MLOGP)	3.66
Log $P_{o/w}$ (SILICOS-IT)	4.00
Consensus Log $P_{o/w}$	3.78

Water Solubility	
Log *S* (ESOL)	-3.90
Solubility	2.77e-02 mg/ml ; 1.26e-04 mol/l
Class	Soluble
Log *S* (Ali)	-4.63
Solubility	5.19e-03 mg/ml ; 2.36e-05 mol/l
Class	Moderately soluble
Log *S* (SILICOS-IT)	-4.50
Solubility	6.95e-03 mg/ml ; 3.16e-05 mol/l
Class	Moderately soluble

Pharmacokinetics	
GI absorption	High
BBB permeant	Yes
P-gp substrate	No
CYP1A2 inhibitor	No
CYP2C19 inhibitor	No
CYP2C9 inhibitor	No
CYP2D6 inhibitor	No
CYP3A4 inhibitor	No
Log K_p (skin permeation)	-4.55 cm/s

Druglikeness	
Lipinski	Yes; 0 violation
Ghose	Yes
Veber	Yes
Egan	Yes
Muegge	Yes
Bioavailability Score	0.55

Medicinal Chemistry	
PAINS	0 alert
Brenk	1 alert: phenol_ester
Leadlikeness	No; 2 violations: MW<250, XLOGP3>3.5
Synthetic accessibility	2.09

Figure S40. Predicted pharmacokinetic parameters of compound **5.**

Molecule 6

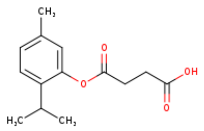

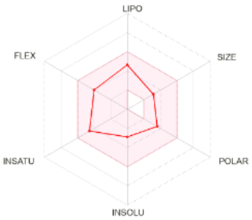

SMILES OC(=O)CCC(=O)Oc1cc(C)ccc1C(C)C

Physicochemical Properties	
Formula	C14H18O4
Molecular weight	250.29 g/mol
Num. heavy atoms	18
Num. arom. heavy atoms	6
Fraction Csp3	0.43
Num. rotatable bonds	6
Num. H-bond acceptors	4
Num. H-bond donors	1
Molar Refractivity	68.87
TPSA	63.60 Å²

Lipophilicity	
Log $P_{o/w}$ (iLOGP)	2.29
Log $P_{o/w}$ (XLOGP3)	2.60
Log $P_{o/w}$ (WLOGP)	2.89
Log $P_{o/w}$ (MLOGP)	2.66
Log $P_{o/w}$ (SILICOS-IT)	2.97
Consensus Log $P_{o/w}$	2.68

Water Solubility	
Log *S* (ESOL)	-2.88
Solubility	3.30e-01 mg/ml ; 1.32e-03 mol/l
Class	Soluble
Log *S* (Ali)	-3.58
Solubility	6.51e-02 mg/ml ; 2.60e-04 mol/l
Class	Soluble
Log *S* (SILICOS-IT)	-3.48
Solubility	8.19e-02 mg/ml ; 3.27e-04 mol/l
Class	Soluble

Pharmacokinetics	
GI absorption	High
BBB permeant	Yes
P-gp substrate	No
CYP1A2 inhibitor	No
CYP2C19 inhibitor	No
CYP2C9 inhibitor	No
CYP2D6 inhibitor	No
CYP3A4 inhibitor	No
Log K_p (skin permeation)	-5.98 cm/s

Druglikeness	
Lipinski	Yes; 0 violation
Ghose	Yes
Veber	Yes
Egan	Yes
Muegge	Yes
Bioavailability Score	0.85

Medicinal Chemistry	
PAINS	0 alert
Brenk	1 alert: phenol_ester
Leadlikeness	Yes
Synthetic accessibility	2.29

Figure S41. Predicted pharmacokinetic parameters of compound **6.**

Molecule 7

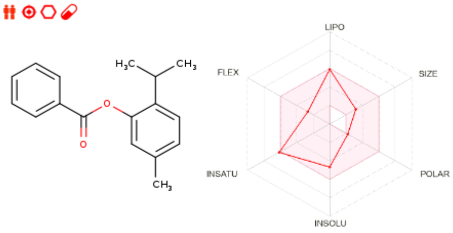

SMILES Cc1ccc(c(c1)OC(=O)c1ccccc1)C(C)C

Physicochemical Properties	
Formula	C17H18O2
Molecular weight	254.32 g/mol
Num. heavy atoms	19
Num. arom. heavy atoms	12
Fraction Csp3	0.24
Num. rotatable bonds	4
Num. H-bond acceptors	2
Num. H-bond donors	0
Molar Refractivity	77.39
TPSA	26.30 Å²

Lipophilicity	
Log $P_{o/w}$ (iLOGP)	3.26
Log $P_{o/w}$ (XLOGP3)	4.82
Log $P_{o/w}$ (WLOGP)	4.34
Log $P_{o/w}$ (MLOGP)	4.43
Log $P_{o/w}$ (SILICOS-IT)	4.49
Consensus Log $P_{o/w}$	4.27

Water Solubility	
Log *S* (ESOL)	-4.66
Solubility	5.61e-03 mg/ml ; 2.20e-05 mol/l
Class	Moderately soluble
Log *S* (Ali)	-5.11
Solubility	2.00e-03 mg/ml ; 7.85e-06 mol/l
Class	Moderately soluble
Log *S* (SILICOS-IT)	-5.82
Solubility	3.87e-04 mg/ml ; 1.52e-06 mol/l
Class	Moderately soluble

Pharmacokinetics	
GI absorption	High
BBB permeant	Yes
P-gp substrate	No
CYP1A2 inhibitor	Yes
CYP2C19 inhibitor	Yes
CYP2C9 inhibitor	No
CYP2D6 inhibitor	Yes
CYP3A4 inhibitor	No
Log K_p (skin permeation)	-4.43 cm/s

Druglikeness	
Lipinski	Yes; 1 violation: MLOGP>4.15
Ghose	Yes
Veber	Yes
Egan	Yes
Muegge	Yes
Bioavailability Score	0.55

Medicinal Chemistry	
PAINS	0 alert
Brenk	1 alert: phenol_ester
Leadlikeness	No; 1 violation: XLOGP3>3.5
Synthetic accessibility	2.18

Figure S42. Predicted pharmacokinetic parameters of compound **7.**

Molecule 8

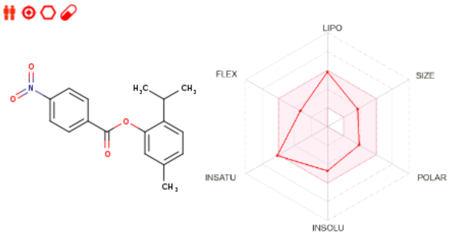

SMILES Cc1ccc(c(c1)OC(=O)c1ccc(cc1)N(=O)=O)C(C)C

Physicochemical Properties	
Formula	C17H17NO4
Molecular weight	299.32 g/mol
Num. heavy atoms	22
Num. arom. heavy atoms	12
Fraction Csp3	0.24
Num. rotatable bonds	5
Num. H-bond acceptors	4
Num. H-bond donors	0
Molar Refractivity	86.21
TPSA	72.12 Å²

Lipophilicity	
Log $P_{o/w}$ (iLOGP)	2.68
Log $P_{o/w}$ (XLOGP3)	4.65
Log $P_{o/w}$ (WLOGP)	4.77
Log $P_{o/w}$ (MLOGP)	3.27
Log $P_{o/w}$ (SILICOS-IT)	2.72
Consensus Log $P_{o/w}$	3.62

Water Solubility	
Log *S* (ESOL)	-4.70
Solubility	5.99e-03 mg/ml ; 2.00e-05 mol/l
Class	Moderately soluble
Log *S* (Ali)	-5.89
Solubility	3.85e-04 mg/ml ; 1.29e-06 mol/l
Class	Moderately soluble
Log *S* (SILICOS-IT)	-5.66
Solubility	6.52e-04 mg/ml ; 2.18e-06 mol/l
Class	Moderately soluble

Pharmacokinetics	
GI absorption	High
BBB permeant	No
P-gp substrate	No
CYP1A2 inhibitor	Yes
CYP2C19 inhibitor	Yes
CYP2C9 inhibitor	No
CYP2D6 inhibitor	No
CYP3A4 inhibitor	No
Log K_p (skin permeation)	-4.82 cm/s

Druglikeness	
Lipinski	Yes; 0 violation
Ghose	Yes
Veber	Yes
Egan	Yes
Muegge	Yes
Bioavailability Score	0.55

Medicinal Chemistry	
PAINS	0 alert
Brenk	2 alerts: nitro_group, phenol_ester
Leadlikeness	No; 1 violation: XLOGP3>3.5
Synthetic accessibility	2.35

Figure S43. Predicted pharmacokinetic parameters of compound **8.**

Molecule 9

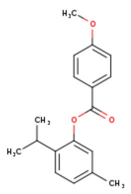

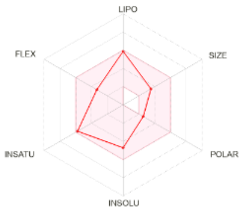

SMILES COc1ccc(cc1)C(=O)Oc1cc(C)ccc1C(C)C

Physicochemical Properties	
Formula	C18H20O3
Molecular weight	284.35 g/mol
Num. heavy atoms	21
Num. arom. heavy atoms	12
Fraction Csp3	0.28
Num. rotatable bonds	5
Num. H-bond acceptors	3
Num. H-bond donors	0
Molar Refractivity	83.88
TPSA	35.53 Å²

Lipophilicity	
Log $P_{o/w}$ (iLOGP)	3.54
Log $P_{o/w}$ (XLOGP3)	4.79
Log $P_{o/w}$ (WLOGP)	4.35
Log $P_{o/w}$ (MLOGP)	4.06
Log $P_{o/w}$ (SILICOS-IT)	4.54
Consensus Log $P_{o/w}$	4.25

Water Solubility	
Log *S* (ESOL)	-4.71
Solubility	5.50e-03 mg/ml ; 1.93e-05 mol/l
Class	Moderately soluble
Log *S* (Ali)	-5.27
Solubility	1.53e-03 mg/ml ; 5.40e-06 mol/l
Class	Moderately soluble
Log *S* (SILICOS-IT)	-5.94
Solubility	3.29e-04 mg/ml ; 1.16e-06 mol/l
Class	Moderately soluble

Pharmacokinetics	
GI absorption	High
BBB permeant	Yes
P-gp substrate	No
CYP1A2 inhibitor	Yes
CYP2C19 inhibitor	Yes
CYP2C9 inhibitor	No
CYP2D6 inhibitor	Yes
CYP3A4 inhibitor	No
Log K_p (skin permeation)	-4.63 cm/s

Druglikeness	
Lipinski	Yes; 0 violation
Ghose	Yes
Veber	Yes
Egan	Yes
Muegge	Yes
Bioavailability Score	0.55

Medicinal Chemistry	
PAINS	0 alert
Brenk	1 alert: phenol_ester
Leadlikeness	No; 1 violation: XLOGP3>3.5
Synthetic accessibility	2.39

Figure S44. Predicted pharmacokinetic parameters of compound **9.**

Molecule 10

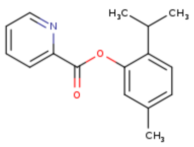

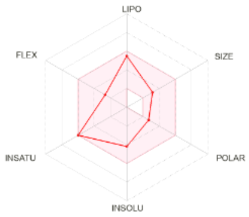

SMILES Cc1ccc(c(c1)OC(=O)c1ccccn1)C(C)C

Physicochemical Properties	
Formula	C16H17NO2
Molecular weight	255.31 g/mol
Num. heavy atoms	19
Num. arom. heavy atoms	12
Fraction Csp3	0.25
Num. rotatable bonds	4
Num. H-bond acceptors	3
Num. H-bond donors	0
Molar Refractivity	75.18
TPSA	39.19 Å²

Lipophilicity	
Log $P_{o/w}$ (iLOGP)	2.75
Log $P_{o/w}$ (XLOGP3)	4.09
Log $P_{o/w}$ (WLOGP)	3.73
Log $P_{o/w}$ (MLOGP)	2.91
Log $P_{o/w}$ (SILICOS-IT)	3.93
Consensus Log $P_{o/w}$	3.48

Water Solubility	
Log *S* (ESOL)	-4.20
Solubility	1.60e-02 mg/ml ; 6.27e-05 mol/l
Class	Moderately soluble
Log *S* (Ali)	-4.62
Solubility	6.15e-03 mg/ml ; 2.41e-05 mol/l
Class	Moderately soluble
Log *S* (SILICOS-IT)	-5.45
Solubility	9.16e-04 mg/ml ; 3.59e-06 mol/l
Class	Moderately soluble

Pharmacokinetics	
GI absorption	High
BBB permeant	Yes
P-gp substrate	No
CYP1A2 inhibitor	Yes
CYP2C19 inhibitor	Yes
CYP2C9 inhibitor	Yes
CYP2D6 inhibitor	Yes
CYP3A4 inhibitor	No
Log K_p (skin permeation)	-4.95 cm/s

Druglikeness	
Lipinski	Yes; 0 violation
Ghose	Yes
Veber	Yes
Egan	Yes
Muegge	Yes
Bioavailability Score	0.55

Medicinal Chemistry	
PAINS	0 alert
Brenk	1 alert: phenol_ester
Leadlikeness	No; 1 violation: XLOGP3>3.5
Synthetic accessibility	2.29

Figure S45. Predicted pharmacokinetic parameters of compound **10.**

Molecule 11

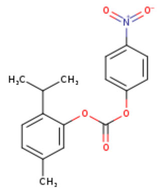

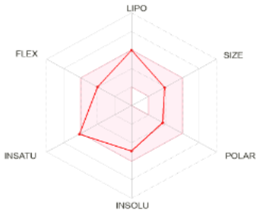

SMILES O=C(Oc1cc(C)ccc1C(C)C)Oc1ccc(cc1)[N+](=O)=[O-]

Physicochemical Properties	
Formula	C17H17NO5
Molecular weight	315.32 g/mol
Num. heavy atoms	23
Num. arom. heavy atoms	12
Fraction Csp3	0.24
Num. rotatable bonds	6
Num. H-bond acceptors	5
Num. H-bond donors	0
Molar Refractivity	84.52
TPSA	66.20 Å²

Lipophilicity	
Log $P_{o/w}$ (iLOGP)	3.12
Log $P_{o/w}$ (XLOGP3)	4.96
Log $P_{o/w}$ (WLOGP)	4.46
Log $P_{o/w}$ (MLOGP)	2.86
Log $P_{o/w}$ (SILICOS-IT)	2.27
Consensus Log $P_{o/w}$	3.54

Water Solubility	
Log *S* (ESOL)	-4.91
Solubility	3.88e-03 mg/ml ; 1.23e-05 mol/l
Class	Moderately soluble
Log *S* (Ali)	-6.09
Solubility	2.57e-04 mg/ml ; 8.16e-07 mol/l
Class	Poorly soluble
Log *S* (SILICOS-IT)	-5.39
Solubility	1.27e-03 mg/ml ; 4.03e-06 mol/l
Class	Moderately soluble

Pharmacokinetics	
GI absorption	High
BBB permeant	Yes
P-gp substrate	No
CYP1A2 inhibitor	Yes
CYP2C19 inhibitor	Yes
CYP2C9 inhibitor	Yes
CYP2D6 inhibitor	No
CYP3A4 inhibitor	No
Log K_p (skin permeation)	-4.70 cm/s

Druglikeness	
Lipinski	Yes; 0 violation
Ghose	Yes
Veber	Yes
Egan	Yes
Muegge	Yes
Bioavailability Score	0.55

Medicinal Chemistry	
PAINS	0 alert
Brenk	3 alerts: nitro_group, phenyl_carbonate, quaternary_nitrogen_3
Leadlikeness	No; 1 violation: XLOGP3>3.5
Synthetic accessibility	2.51

Figure S46. Predicted pharmacokinetic parameters of compound **11**

Molecule 12

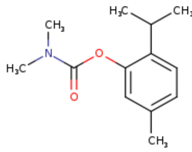

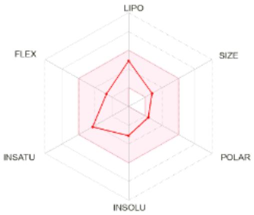

SMILES Cc1ccc(c(c1)OC(=O)N(C)C)C(C)C

Physicochemical Properties	
Formula	C13H19NO2
Molecular weight	221.30 g/mol
Num. heavy atoms	16
Num. arom. heavy atoms	6
Fraction Csp3	0.46
Num. rotatable bonds	4
Num. H-bond acceptors	2
Num. H-bond donors	0
Molar Refractivity	65.58
TPSA	29.54 Å²

Lipophilicity	
Log $P_{o/w}$ (iLOGP)	3.03
Log $P_{o/w}$ (XLOGP3)	3.01
Log $P_{o/w}$ (WLOGP)	3.18
Log $P_{o/w}$ (MLOGP)	2.92
Log $P_{o/w}$ (SILICOS-IT)	2.39
Consensus Log $P_{o/w}$	2.91

Water Solubility	
Log *S* (ESOL)	-3.12
Solubility	1.67e-01 mg/ml ; 7.55e-04 mol/l
Class	Soluble
Log *S* (Ali)	-3.30
Solubility	1.12e-01 mg/ml ; 5.07e-04 mol/l
Class	Soluble
Log *S* (SILICOS-IT)	-3.42
Solubility	8.51e-02 mg/ml ; 3.85e-04 mol/l
Class	Soluble

Pharmacokinetics	
GI absorption	High
BBB permeant	Yes
P-gp substrate	No
CYP1A2 inhibitor	No
CYP2C19 inhibitor	Yes
CYP2C9 inhibitor	No
CYP2D6 inhibitor	No
CYP3A4 inhibitor	No
Log K_p (skin permeation)	-5.51 cm/s

Druglikeness	
Lipinski	Yes; 0 violation
Ghose	Yes
Veber	Yes
Egan	Yes
Muegge	Yes
Bioavailability Score	0.55

Medicinal Chemistry	
PAINS	0 alert
Brenk	0 alert
Leadlikeness	No; 1 violation: MW<250
Synthetic accessibility	2.12

Figure S47. Predicted pharmacokinetic parameters of compound **12**

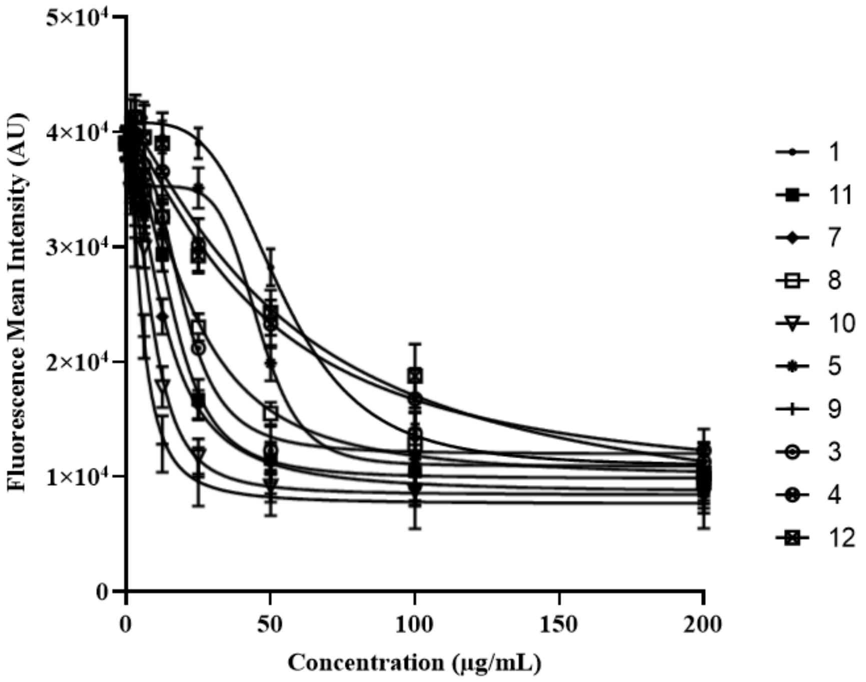

Figure S48, IC_{50} measurements across replicates of *Leishmania amazonensis*.

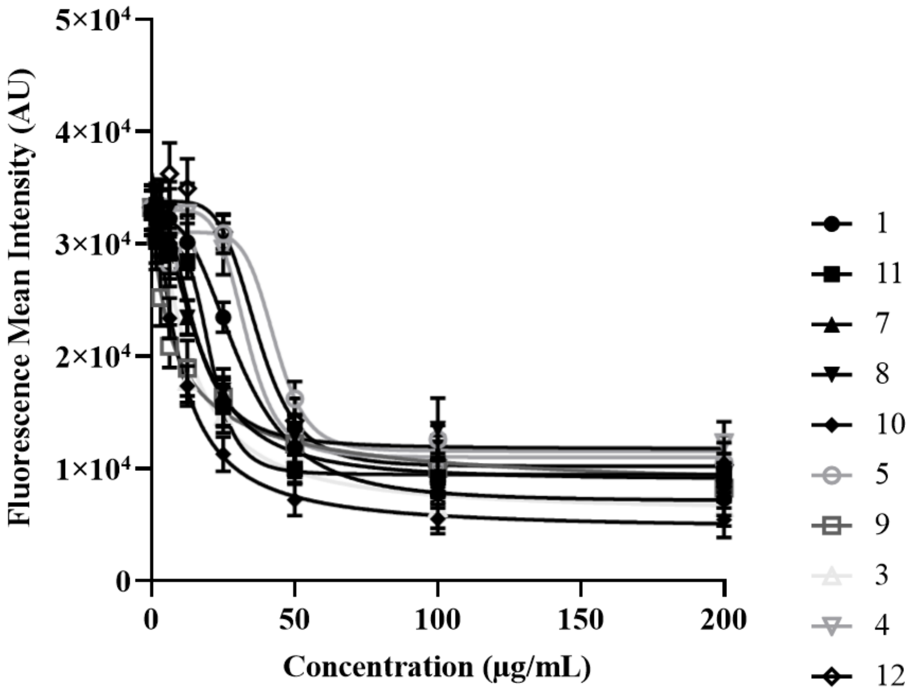

Figure S49, IC_{50} measurements across replicates of *Trypanosoma cruzi*.

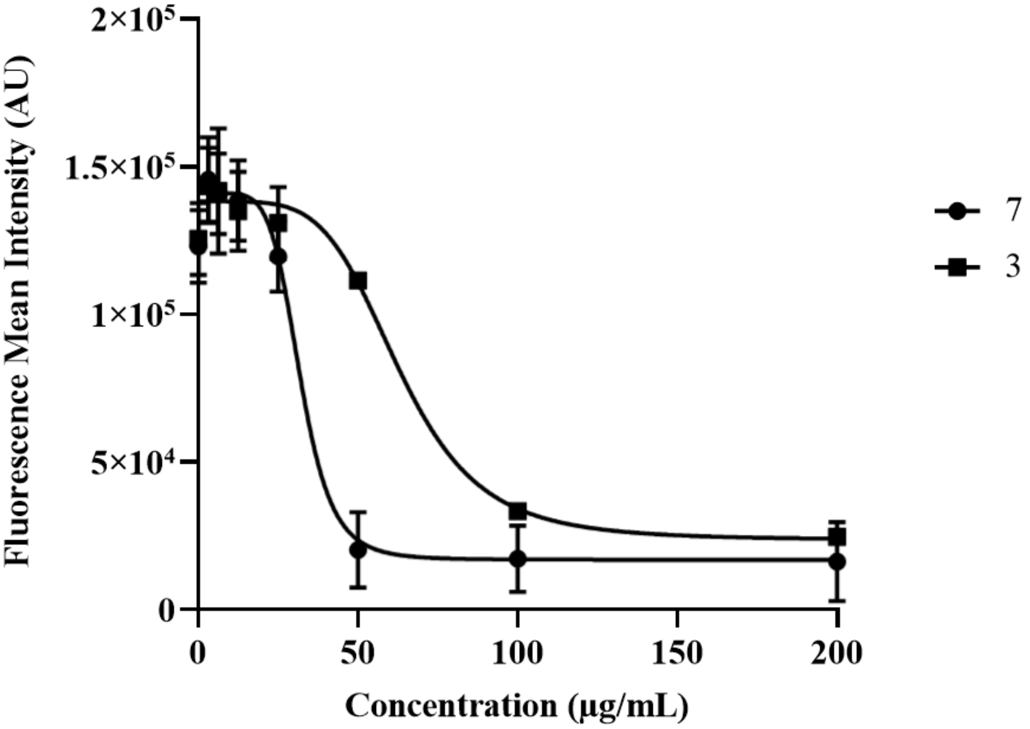

Figure S50, curves for CC_{50} measurements.
